# Dapagliflozin Prevents Heart Failure With Preserved Ejection Fraction by Inhibiting the Sodium/Hydrogen Exchanger

**DOI:** 10.1016/j.jacbts.2026.101698

**Published:** 2026-09-18

**Authors:** Man Liu, Hong Liu, Eunji Kim, Gyeoung-Jin Kang, Mitchell C. Neumann, Madeline Johnson, Ruthvika Murikinati, Samuel C. Dudley

**Affiliations:** Cardiovascular Division, Department of Medicine, The Lillehei Heart Institute, University of Minnesota at Twin Cities, Minneapolis, Minnesota, USA

**Keywords:** cardiac diastolic dysfunction, inflammation, mitochondrial dysfunction, Mg^2+^, NHE1, TRPM7

## Abstract

Sodium-glucose cotransporter 2 (SGLT2) inhibitors have shown protective effects against heart failure with preserved ejection fraction (HFpEF), but SGLT2 is not expressed significantly in the heart. Here, we investigated the mechanism by which the SGLT2 inhibitor dapagliflozin (Dapa) alters HypoMg-associated HFpEF. HypoMg was induced by a low-Mg diet in mice or in a human cardiomyocyte cell line. Three weeks of Dapa treatment prevented HypoMg-induced HFpEF in mice. In RL-14 cardiomyocytes, sodium-hydrogen exchanger 1 (NHE1) overexpression or activation mimicked the cellular effects of HypoMg. Dapa reversed these changes. Dapa prevented cardiac HFpEF by inhibiting cardiomyocyte NHE1 activity and suppressing macrophage activation.

## Highlights

• SGLT2 inhibitors are used for HFpEF, but there is no SGLT2 in heart.
• Dapagliflozin prevented hypomagnesemia-induced diastolic dysfunction.
• Dapagliflozin had identical effects to NHE1 inhibition on reversing these changes.

## Visual Abstract

**Figure:**
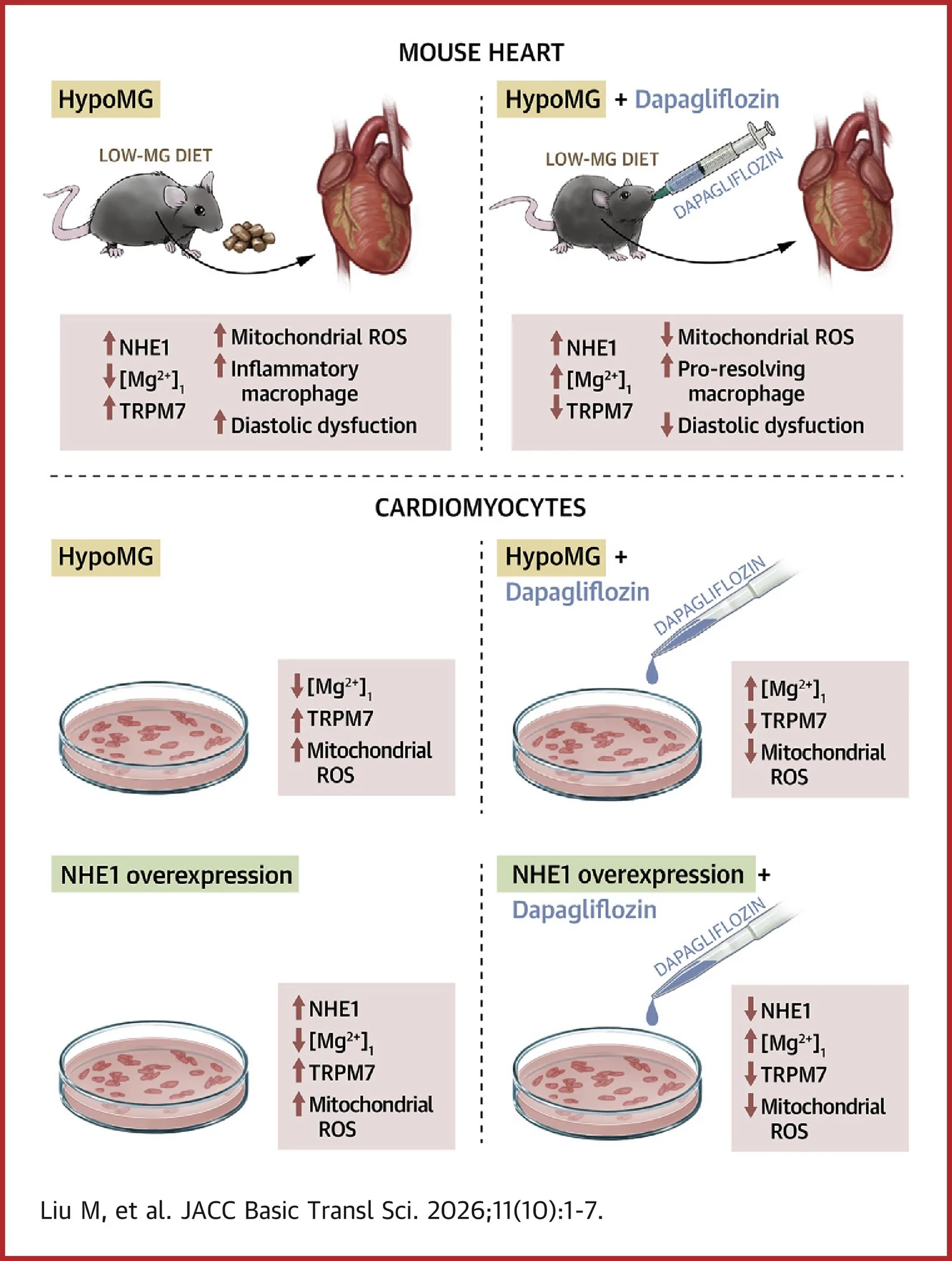

In the United States, ∼6.7 million people have heart failure (HF), and the number is increasing yearly to reach more than 11 million by 2050.^1^^,^^2^ Approximately 50% of HF cases are heart failure with preserved ejection fraction (HFpEF).^3^^,^^4^ The 5-year mortality rate of HFpEF is 55% to 74%, which is similar to heart failure with reduced ejection fraction (HFrEF).^5^^,^^6^ Underlying most HFpEF is cardiac diastolic dysfunction (DD) that is characterized by the reduced ability of the left ventricle to relax and fill adequately with blood. DD progresses to HFpEF in a significant percentage of patients.^7^, ^8^, ^9^ Many drugs that are approved to treat systolic HF fail to show efficacy against DD or HFpEF.^3^^,^^10^^,^^11^ There are limited specific treatments for HFpEF^12^, ^13^, ^14^ because of poor understanding of the underlying pathophysiology.^7^^,^^15^^,^^16^ Sodium-glucose cotransporter 2 (SGLT2) inhibitors (ie flozins) such as dapagliflozin (Dapa, brand name Farxiga) and empagliflozin (brand name Jardiance) are among the few treatments that are approved for use in HFpEF, showing reduced risks of hospitalization for patients with HFpEF.^12^^,^^13^ Nevertheless, SGLT2 is not expressed in heart,^17^^,^^18^ causing uncertainty about the mechanism of actions of SGLT2 inhibitors.

Diabetes is among many risk factors for HFpEF that include obesity, aging, metabolic syndrome, and chronic kidney diseases.^19^^,^^20^ Our previous studies have shown that hypomagnesemia (HypoMg, serum Mg^2+^ <0.8 mmol/L) and subsequent mitochondrial oxidative stress cause diabetic cardiac DD and HFpEF.^21^^,^^22^ Suggesting a link between Mg^2+^ and HFpEF, patients with HFpEF and low serum magnesium (Mg^2+^) levels have significantly and independently higher occurrence of future HF-related events than patients with HFpEF but higher serum Mg^2+^ levels.^23^ Macrophage-mediated cardiac inflammation and cardiomyocyte mitochondrial oxidative stress play important roles in the pathogenesis of both diabetes-mediated and HypoMg-induced HFpEF.^21^^,^^22^^,^^24^^,^^25^ HypoMg results in overexpression of the Mg^2+^ channel transient receptor potential melastatin 7 channel (TRPM7).^26^, ^27^, ^28^ TRPM7 also has an α-kinase domain (aa1597-1821)^29^, ^30^, ^31^ that regulates protein transcription^32^^,^^33^ and phosphorylation.^29^^,^^34^^,^^35^ The chanzyme is essential for early cardiogenesis, cardiac repolarization, automaticity, and rhythmicity.^36^^,^^37^ Inhibition of the TRPM7 kinase function prevents HypoMg-mediated cardiac DD by preventing inflammation and mitochondrial oxidative stress.^25^^,^^28^ Mg^2+^ supplementation or repletion reverses HFpEF by increasing serum Mg^2+^ levels and decreasing TRPM7, inflammation, and mitochondrial reactive oxygen species (mitoROS).^21^^,^^22^

SGLT2 inhibitors have been shown to increase serum Mg^2+^ levels of patients with or without diabetes^38^^,^^39^ and improve mitochondrial redox and function.^40^, ^41^, ^42^, ^43^ Among the commonly used SGLT2 inhibitors, Dapa shows the strongest effect on elevating serum Mg^2+^levels^38^^,^^44^ and inhibits oxidative stress and inflammation in diabetic mouse kidney.^45^ In this work, we investigated the effects of Dapa on macrophage inflammatory activation and cardiomyocyte mitochondrial dysfunction in a HypoMg HFpEF model that should minimize any confounding effects of Dapa on serum glucose.

## Methods

Detailed methods are described in the Supplemental Appendix. All chemicals, antibodies, and supplies were purchased from Sigma-Aldrich Inc unless otherwise stated.

### Study approval

Animal care and interventions were undertaken in accordance with the National Institutes of Health Guide for the Care and Use of Experimental Animals, and the animal protocol (IACUC-2303-40873A and IACUC-2601-43586A) was approved by the Institutional Animal Care and Use Committees of the University of Minnesota.

### Sex as a biological variable

Equal numbers of both sexes of mice were studied in control and HypoMg + Dapa group, whereas mainly male mice were tested in the HypoMg group because of excess mortality in the female mice exposed to HypoMg.^22^

### Animal treatment

C57BL/6J mice (The Jackson Laboratory, Strain #000664) were randomly assigned to different groups at 10 weeks of age. Control mice were fed with a normal chow (containing 2 g/kg Mg^2+^, Envigo Teklad global 18% protein rodent diet 2018; Envigo Teklad Diets) for 6 weeks. HypoMg mice were fed with a low-Mg diet (containing 15-30 mg/kg Mg^2+^, TD.93106, Envigo Teklad Diets) for 6 weeks, with distilled and deionized water to control any possible Mg^2+^ intake from drinking water. The low-Mg diet was chosen based on previous animal studies.^22^^,^^28^^,^^46^ Dapa (Advanced ChemiBlocks Inc) was given to approximately half of the HypoMg mice by oral gavage at 1.5 mg/kg daily for 3 weeks, the approximate equivalent of 21 months of human dosing, starting at the fourth week of the low-Mg diet. The dose, treatment timing, and length of Dapa were modified from other animal studies with Dapa.^47^^,^^48^

### Cell treatment

Human fetal RL-14 cardiomyocytes (ATCC PTA-1499; RRID: CVCL_4U19) were cultured with normal Dulbecco’s modified Eagle’s medium/F-12 (Thermo Fisher Scientific #11995065 with 0.81 mM Mg^2+^) or a low-Mg medium (modified from Thermo Fisher Scientific #11995065 with 0.04 mM Mg^2+^) in the presence or absence of Dapa (5 μmol/L; Advanced ChemBlocks Inc), rosmarinic acid (10 ng/mL; Cayman Chemical), cariporide (10 μmol/L; MedChemExpress LLC), or TG100-115 treatment (3-[2,4-diamino-7-(3-hydroxyphenyl)pteridin-6-yl]phenol, 10 mmol/L; AmBeed, Bepharm Scientific Inc) at 37 °C in a humidified atmosphere containing 5% CO_2_ and 95% O_2_ for 48 hours. These concentrations were based on previous reports.^49^, ^50^, ^51^, ^52^

### SGLT2 detection in mouse ventricle

Reverse-transcriptase polymerase chain reaction (RT-PCR) and mass spectrometry were used to detect expression of SGLT2 in the mouse ventricles. For RT-PCR, total RNA was isolated from mouse ventricular tissue using the QIAGEN RNeasy Kit (Qiagen) according to the manufacturer's protocol. RNA concentration and purity were assessed using a NanoDrop spectrophotometer (Thermo Fisher Scientific) by measuring the 260/280 absorbance ratio. Only samples with a 260/280 ratio between 1.8 and 2.1 were considered high purity and used for subsequent complementary DNA (cDNA) synthesis. First-strand cDNA was synthesized from 1 μg of total RNA using the LunaScript RT SuperMix Kit (New England Biolabs). Quantitative polymerase chain reaction (PCR) was performed using PowerUp SYBR Green Master Mix (Thermo Fisher Scientific) on a 7500 Fast Real-Time PCR System (Thermo Fisher Scientific). The primers used for mouse SGLT2 (*Slc5a2*) were as follows: forward, 5′-ATGGAGCAACACGTAGAGGC-3′ and reverse, 5′-ATGACCAGCAGGAAATAGGCA-3′. Gene expression was normalized to the housekeeping gene *Gapdh* (forward: 5′-AGTGTTTCCTCGTCCCGTAG -3′, reverse: 5′-GCCGTGAGTGGAGTCATACT-3′) and relative expression was calculated using the 2^ˆ^-ΔCt method. For mass spectrometry, we ran protein samples immunoprecipitated with SGLT2 antibody (Santa Cruz) on the sodium dodecyl sulfate–polyacrylamide gel electrophoresis (SDS-PAGE) gel, stained the gel with EZBlue Gel Staining reagent, and cut and sent the gel to the Center for Metabolomics and Proteomics, University of Minnesota for mass spectrometry.

### Sodium-hydrogen exchanger 1 overexpression in RL-14 cardiomyocytes

To generate the sodium-hydrogen exchanger 1 (NHE1) overexpression construct, the full-length cDNA of human *SLC9A1* was amplified by PCR. The resulting PCR product was subcloned into the pcDNA3.1 mammalian expression vector using the HindIII and XhoI restriction sites. For transfection, RL-14 cells were seeded and transfected with the SLC9A1-pcDNA3.1 plasmid using the FuGENE 6 Transfection Reagent (Promega) in accordance with the manufacturer's protocol.

### Cardiac DD evaluation

Noninvasive echocardiography was conducted on mice using a Vevo 2100 ultrasound system (VisualSonics) to measure the ratio between mitral peak early filling velocity E and longitudinal tissue velocity of the mitral anterior annulus e’ (E/e’) as we have done previously.^21^^,^^22^^,^^28^ Mice were anesthetized with 1% to 2% isoflurane in oxygen at 1 L/min with the body temperature and heart rate maintained at 37 to 38 °C and above 400 beats/min, respectively, during the procedure. B-mode images along the left ventricular parasternal long axis and then M-mode images at the mid-papillary level were obtained to calculate ejection fraction and chamber size. To evaluate diastolic function, mitral inflow velocity E and longitudinal tissue velocity of the mitral anterior annulus e’ were assessed in the subcostal 4-chamber view by pulsed-wave and tissue Doppler imaging to calculate E/e’. The absolute value of E/e’ was used, following the usual convention. Measurements were averaged from 5 consecutive beats during expiration.

### Serum and urine Mg^2+^ levels

Serum and urine Mg^2+^ levels were measured with Magnesium Assay Kit as done previously.^22^^,^^28^

### Cardiac macrophage phenotyping

Flow cytometry was performed to study cardiac macrophage phenotype in mouse ventricles with isolated cardiac interstitial cells and fluorescent antibodies of different types of macrophages as we have done previously.^24^ Isolated non-cardiomyocyte interstitial cells were pre-gated on CD11b^+^F4/80^+^ as cardiac macrophages and were further divided into subsets based on their expression of monocyte chemoattractant protein-1 (MCP-1) receptor C-C chemokine receptor 2 (CCR2) and T-cell immunoglobulin and mucin domain-containing 4 (Timd4). Proinflammatory macrophages were labeled with CCR2 but not Timd4 (CCR2^+^/Timd4^−^) in the total CD11b^+^F4/80^+^-labeled macrophages. Proresolving macrophages were labeled with Timd4 but not CCR2 (CCR2^−^/Timd4^+^) in the total CD11b^+^F4/80^+^-labeled macrophages.

### Western blotting for protein assay

Mouse ventricle tissues and cultured cell line lysates were used for protein assays with standard immunoprecipitation. For *S*-glutathionylation of cardiac myosin binding protein C (*S*-Glu-cMyBPC), protein lysates prepared from mouse ventricles were solubilized in a nonreducing 2× Laemmli buffer (Bio-Rad) as done previously.^21^^,^^22^ Without adding reducing agent and sample heating, proteins were separated on SDS-PAGE gels for standard Western blotting. For other protein targets, 2-mercaptoethanol (2.5% final) was applied to the protein lysates and samples were heated (95 °C for 5 minutes) for denaturation. Samples were then separated on SDS-PAGE gels and transferred to 0.2 μm polyvinyl difluoride membranes. After blocking with 5% nonfat milk or 5% bovine serum albumin for 1 hour at room temperature, the membranes were incubated with the corresponding primary antibodies overnight at 4 °C, followed by incubation with appropriate horseradish peroxidase–conjugated secondary antibodies (Bio-Rad Laboratories) for 1 hour at room temperature. Protein bands were visualized by chemiluminescence detection, and the band optical density was analyzed with Image Lab 6.0.0 Software (Bio-Rad Laboratories). Protein levels were normalized first by the loading controls (α-tubulin, β-actin, vinculin, or GAPDH) and then by the control group.

### Fluorescent confocal imaging

Intracellular free Mg^2+^ levels, mitoROS, and intracellular pH were measured with Thermo Fisher Scientific fluorescent probes Mag-Furo 4 AM (1 μmol/L), MitoSox Red (1 μmol/L), and pHrodo Green AM (5 μmol/L), respectively, by confocal imaging, following the manufacturer's guides, as described before.^21^^,^^22^^,^^28^^,^^53^

### Statistics

Data are presented as the mean ± SD. For the dot-bar plots, the bar shows the mean values and the error bars the SD values. One-way analysis of variance with Tukey's multiple comparisons test or unpaired *t*-test was used where appropriate. The Shapiro-Wilk test was performed to confirm that the data were normally distributed. All statistical analyses were performed with GraphPad Prism 10.6 (GraphPad Software). A *P* value < 0.05 was considered statistically significant.

### Data availability

All data associated with this study can be found in the Supplemental Supporting Data Values file.

## Results

### Dapa prevented HypoMg-induced HFpEF

We used a HypoMg-induced cardiac DD/HFpEF mouse model, which has significantly increased E/e’ and left ventricular end-diastolic pressure (LVEDP: 11.9 ± 7.0 mm Hg for HypoMg vs 3.9 ± 1.6 mm Hg for control, *P* = 0.005), as reported in our previous work,^28^ unchanged left ventricular ejection fraction (48.3% ± 5.1% for HypoMg vs 50.0% ± 4.2% for control, *P* = 0.71, Supplemental Figure 1A), normal ratio of height weight to tibia length or heart weight to body weight (Supplemental Figure 1B), left ventricular posterior wall thickness,^28^ systolic blood pressure (104.8 ± 7.8 mm Hg for HypoMg vs 98.6 ± 6.1 mm Hg for control, *P* = 0.17) and diastolic blood pressure (77.5 ± 7.2 mm Hg for HypoMg vs 70.4 ± 5.9 mm Hg for control, *P* = 0.11), and homeostatic model assessment of insulin resistance (6.6 ± 5.1 for HypoMg vs 4.5 ± 4.6 for control, *P* = 0.30). Dapa treatment prevented HypoMg-induced cardiac DD with decreased E/e’ (16.5 ± 2.4 of HypoMg + Dapa vs 20.6 ± 4.2 of HypoMg, *P* = 0.019; vs 15.8 ± 2.0 of control, *P* = 0.90; control vs HypoMg, *P* < 0.001; Figures 1A and 1B: representative images of echocardiography with e’ and a’ peaks) obtained from echocardiography. At the molecular level, a cardiac DD biomarker,^21^^,^^54^^,^^55^ the ratio of an oxidized form of cardiac myosin binding protein C (cMyBPC) – *S*-glutathionylated cMyBPC (*S*-Glu-cMyBPC) to total cMyBPC, was elevated in DD (1.5 ± 0.2-fold of control, *P* = 0.012) and was decreased markedly by Dapa treatment (0.13 ± 0.05-fold of control, *P* < 0.001 vs HypoMg and *P* < 0.001 vs control; Figure 1B). In addition, the total cMyBPC levels were significantly augmented by Dapa treatment (2.7 ± 0.8-fold vs control, *P* = 0.002; Figure 1C).

**Figure 1:**
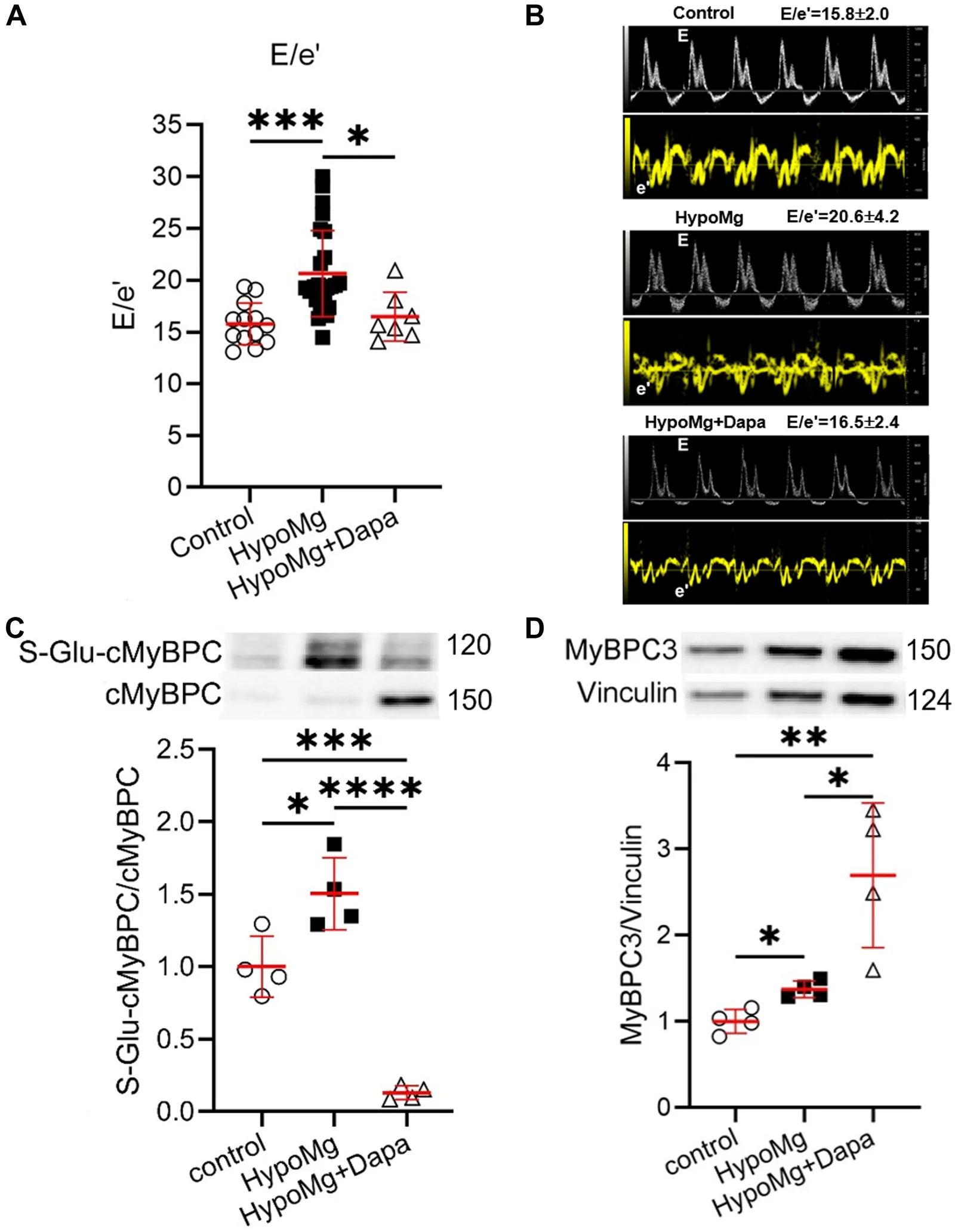
Oral Dapa Prevented HypoMg-Induced HFpEF (A) Cardiac diastolic function was evaluated with E/e’ from echocardiography. Seven to 25 mice were tested per group. (B) Representative mouse echocardiographic images of pulsed-wave Doppler (marked E waves) and tissue Doppler (marked e’ waves) and the mean ± SD of E/e’ for each group. (C) The ratio of *S*-Glu-cMyBPC/total cMyBPC was significantly decreased by Dapa treatment. (D) Total cMyBPC was significantly increased by Dapa treatment. In (C) and (D), 4 mice were tested per group; protein molecular weight was labeled in kDa. Dapa treatment started after 3 weeks of HypoMg diet in half of the HypoMg group and lasted for 3 weeks. Red bars in figures are mean ± SD. One-way analysis of variance with Tukey's multiple comparisons test was used for statistical analysis, ∗*P* < 0.05, ∗∗*P* < 0.01, ∗∗∗*P* < 0.001. Dapa = dapagliflozin; E/e’ = the ratio between mitral peak early filling velocity E and longitudinal tissue velocity of the mitral anterior annulus e’; HypoMg = hypomagnesemia; MyBPC3 = cardiac myosin binding protein C 3; *S*-Glu-cMyBPC = *S*-glutathionylated cardiac myosin binding protein C.

### Dapa elevated serum Mg^2+^

Similar to our previous reports,^22^^,^^25^^,^^28^ the low-Mg diet induced HypoMg with serum Mg^2+^ levels dropping from 1.3 ± 0.2 mmol/L in control mice to 0.6 ± 0.2 mmol/L in HypoMg mice (*P* < 0.001). Dapa treatment in HypoMg mice normalized serum Mg^2+^ levels (*P* < 0.001 vs HypoMg and *P* = 0.75 vs control, Figure 2A). Explaining in part the increased serum level, Dapa resulted in reduced urine Mg^2+^ extrusion (urine Mg^2+^ in mmol/L: 13.9 ± 2.7 of control vs 0.2 ± 0.1 of HypoMg, *P* < 0.001; 0.1 ± 0.0 of HypoMg + Dapa, *P* = 0.006 vs HypoMg and *P* < 0.001 vs control; Figure 2B).

**Figure 2:**
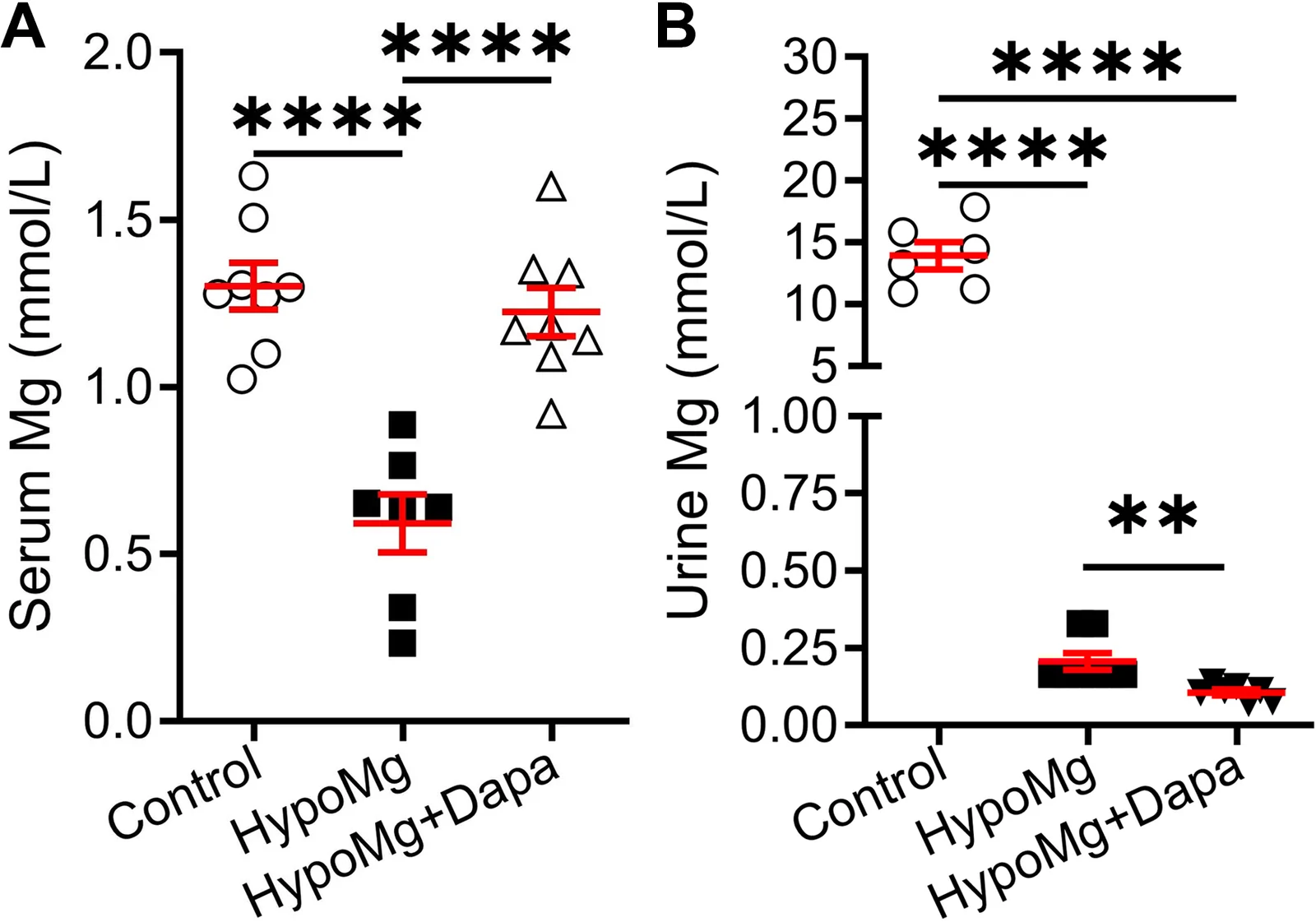
Dapa Prevented the Low-Mg Diet Induced HypoMg (A) Serum Mg^2+^ levels. (B) Urine Mg^2+^ extrusion. Dapa treatment was the same as in Figure 1. Seven to 8 mice were tested for each group. Red bars in figures are mean ± SD. One-way analysis of variance with Tukey's multiple comparisons test was used for statistical analysis. ∗∗*P* < 0.01, ∗∗∗∗*P* < 0.001. Abbreviations as in Figure 1.

### Dapa prevented TRPM7 overexpression and subsequent mitochondrial oxidative stress

Our recent study shows that HypoMg elevates TRPM7 expression and that increased TRPM7 kinase function contributes to mitochondrial oxidative stress and inflammation in the heart^25^^,^^28^ and brain.^56^ Here, we found that HypoMg-induced TRPM7 overexpression (1.4 ± 0.2-fold of control, *P* = 0.030) was normalized by Dapa treatment (1.0 ± 0.2-fold of control, *P* = 0.015 vs HypoMg and *P* = 0.92 vs control; Figure 3A). As expected for a decrease in TRPM7,^28^ HypoMg-induced excess mitoROS (1.6 ± 0.2-fold of control, *P* < 0.001 vs control, Figure 3B) was diminished by Dapa treatment (0.9 ± 0.1-fold of control, *P* < 0.001 vs HypoMg and *P* > 0.99 vs control). Mitochondrial NADPH oxidase 4 (NOX4) also has been shown to contribute to reactive oxygen species overproduction,^45^ and this too was normalized by Dapa (Figure 3C). The HypoMg-induced reduction of the mitochondrial antioxidant manganese superoxide dismutase (0.8 ± 0.1-fold of control, *P* = 0.049 vs control, Supplemental Figure 2) was not improved by Dapa treatment (0.5 ± 0.1-fold of control, *P* = 0.10 vs HypoMg and *P* = 0.002 vs control), however.

**Figure 3:**
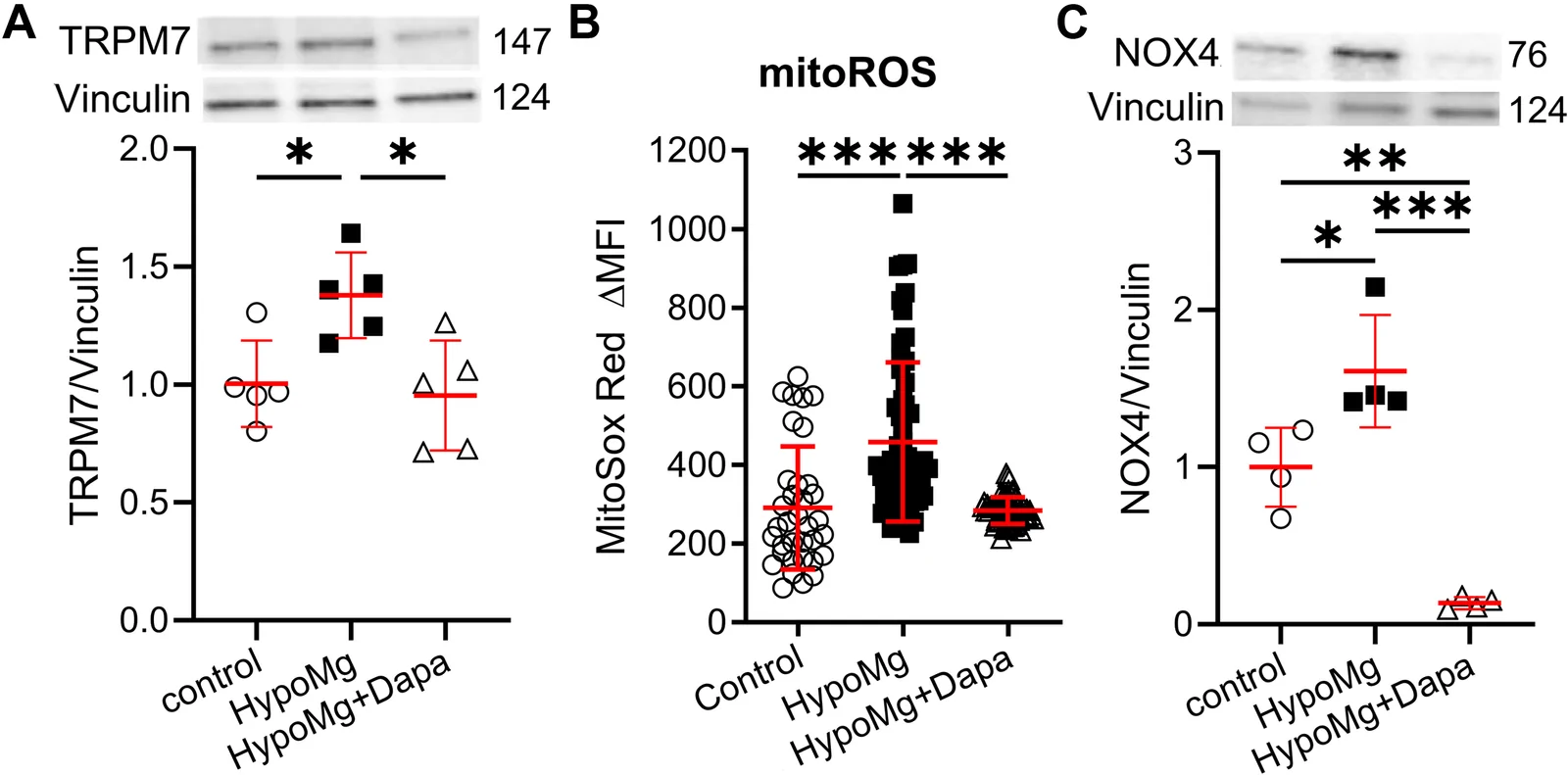
Dapa Prevented HypoMg-Induced Elevation of TRPM7 Expression, MitoROS Overproduction, and (C) NOX4 Expression in Ventricles (A) TRPM7 expression. (B) mitoROS overproduction. (C) NOX4 expression. The mouse treatments were the same as in Figure 1. Four to 5 mice were tested per group in (A) and (C) and protein molecular weight was labeled in kDa. MitoROS levels of isolated cardiomyocytes were measured with MitoSox Red fluorescence by confocal imaging (*Z*-stack); 35 to 62 cardiomyocytes from 3 mice were tested per group. Red bars in figures are mean ± SD. One-way analysis of variance with Tukey's multiple comparisons test was used for statistical analysis, ∗*P* < 0.05, ∗∗*P* < 0.01, ∗∗∗*P* < 0.001. ΔMFI = the fluorescent intensity difference between a cell and the background in the same image; mitoROS = mitochondrial ROS; NOX4 = NADPH oxidase 4; TRPM7 = transient receptor potential melastatin 7; other abbreviations as in Figure 1.

### Dapa alleviated HypoMg-induced inflammation by enhancing resolution of inflammation

Our recent studies show that TRPM7 kinase activates inflammation in both the heart and brain of HypoMg mice.^25^^,^^56^ Consistent with this, we observed HypoMg-induced ventricular inflammation (Figures 4A to 4F) with increased expression of MCP-1 (1.5 ± 0.2-fold of control, *P* = 0.048), cardiac macrophage infiltration as measured by marker CD68 (1.5 ± 0.2-fold of control, *P* = 0.008), NLR family pyrin domain-containing protein 3 (NLRP3, 3.2 ± 0.9-fold of control, *P* = 0.002), mature interleukin (IL)-1β (1.5 ± 0.2-fold of control, *P* = 0.018), and IL-18 (3.5 ± 1.3-fold of control, *P* = 0.003). These changes were alleviated by Dapa treatment (Figures 4A to 4F).

**Figure 4:**
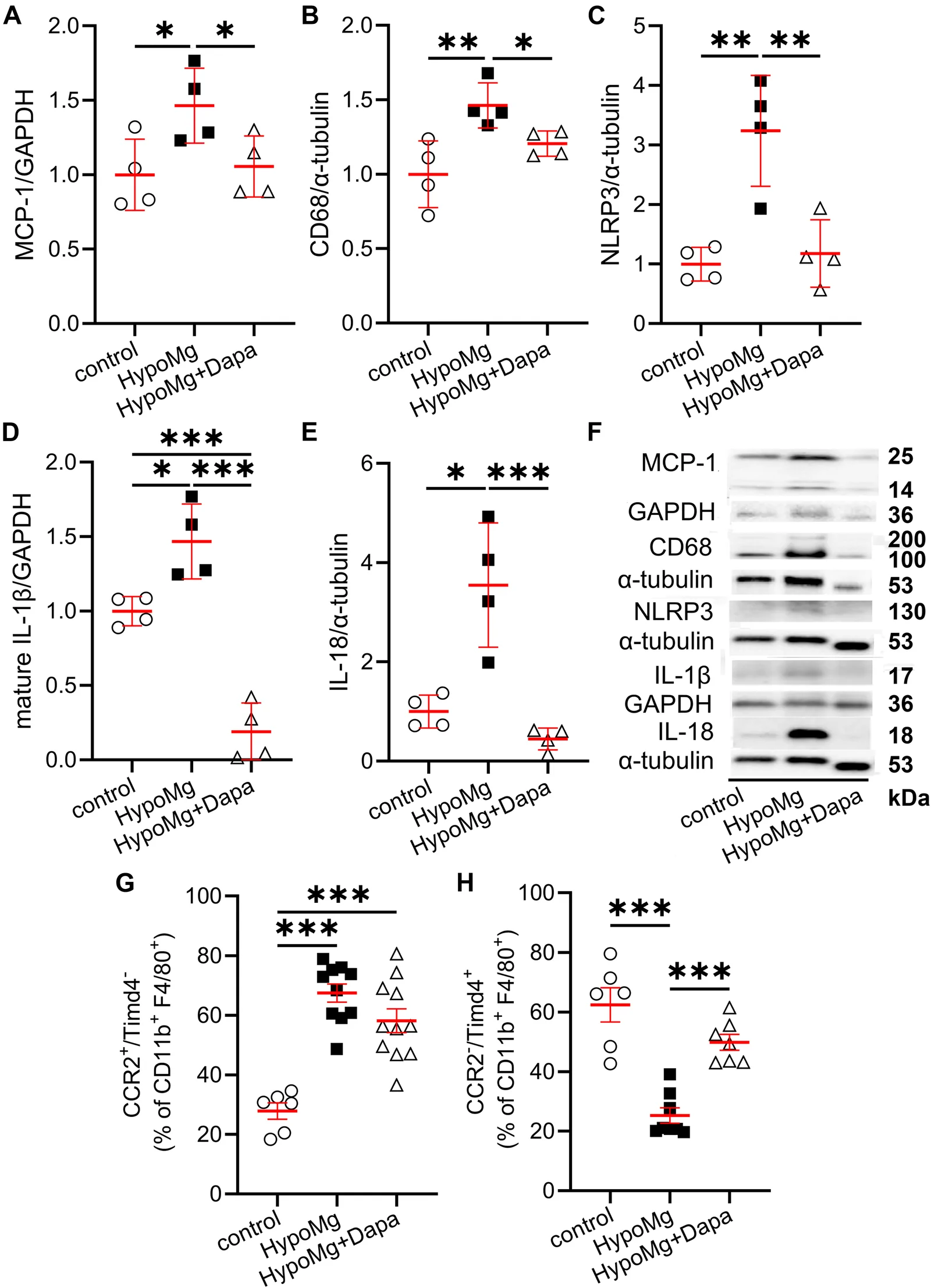
Dapa Treatment Alleviated HypoMg-Induced Cardiac Inflammation Protein expression of (A) MCP-1, (B) CD68, (C) NLRP3, (D) IL-1β, and (E) IL-18 in the mouse ventricular tissues. (F) Representative Western blot bands with protein molecular weight in kDa. (G) Proinflammatory macrophages labeled with CCR2 but not Timd4 (CCR2^+^/Timd4^−^) in the total CD11b^+^F4/80^+^-labeled macrophages. (H) Proresolving macrophages labeled with Timd4 but not CCR2 (CCR2^−^/Timd4^+^) in the total CD11b^+^F4/80^+^-labeled macrophages. The mouse treatments were the same as in Figure 1. Four mice were tested for each group in (A)-(E). Six to 7 mice were tested per group in (G) and (H). Red bars in figures are mean ± SD. One-way analysis of variance with Tukey's multiple comparisons test was used for statistical analysis, ∗*P* < 0.05, ∗∗*P* < 0.01, ∗∗∗*P* < 0.001. CD68 = cluster of differentiation 68; CCR2^+^ = C-C chemokine receptor 2; IL-1β = interleukin-1β; IL-18 = interleukin-18; MCP-1 = monocyte chemoattractant protein-1; NLRP3 = NLR family pyrin domain-containing protein 3; Timd4 = T-cell immunoglobulin and mucin domain-containing 4; other abbreviations as in Figure 1.

Cardiac macrophage phenotype was evaluated with flow cytometry. Macrophages were divided into subsets based on the expression of MCP-1 receptor CCR2 and Timd4. CCR2^+^/Timd4^−^ cells are thought to be proinflammatory macrophages, and CCR2^−^/Timd4^+^ cells are proresolving macrophages.^57^^,^^58^ Explaining the reduction in inflammatory cytokines, Dapa augmented proresolving macrophages (49.8% ± 7.1% of total macrophages in ventricles of HypoMg + Dapa vs 25.3% ± 7.3% of HypoMg, *P* < 0.001; vs of 62.4% ± 14.1% control, *P* = 0.073; *P* < 0.001 for HypoMg vs control; Figures 4G and 4H).

### Dapa increased intracellular Mg^2+^ levels and reduced cardiomyocyte oxidative stress

We used a human fetal ventricular cardiomyocyte cell line RL-14^59^ to test potential mechanisms for the Dapa effect. Applying Dapa (5 μmol/L) to RL-14 cardiomyocytes cultured in low-Mg medium (containing 0.04 mmol/L Mg^2+^) for 48 hours normalized the HypoMg-induced reduction of intracellular Mg^2+^ levels (1.0 ± 1.0 for control; 0.5 ± 0.4 for HypoMg, *P* = 0.031 vs control; 1.5 ± 1.0 for HypoMg + Dapa, *P* = 0.042 vs control and *P* < 0.001 vs HypoMg; Figure 5A). As expected for an increase in intracellular Mg^2+^,^21^^,^^22^^,^^28^ Dapa prevented the elevation of TRPM7 protein expression (Figure 5B, HypoMg: 2.3 ± 0.5-fold of control, *P* = 0.006; HypoMg + Dapa: 1.4 ± 0.1-fold of control, *P* = 0.29 vs control and *P* = 0.035 vs HypoMg) and mitoROS overproduction (Figure 5C, 25.7 ± 6.2 for control; 41.6 ± 18.1 for HypoMg, *P* < 0.001 vs control; 25.2 ± 10.7 for HypoMg + Dapa, *P* = 0.93 vs control and *P* < 0.001 vs HypoMg).

**Figure 5:**
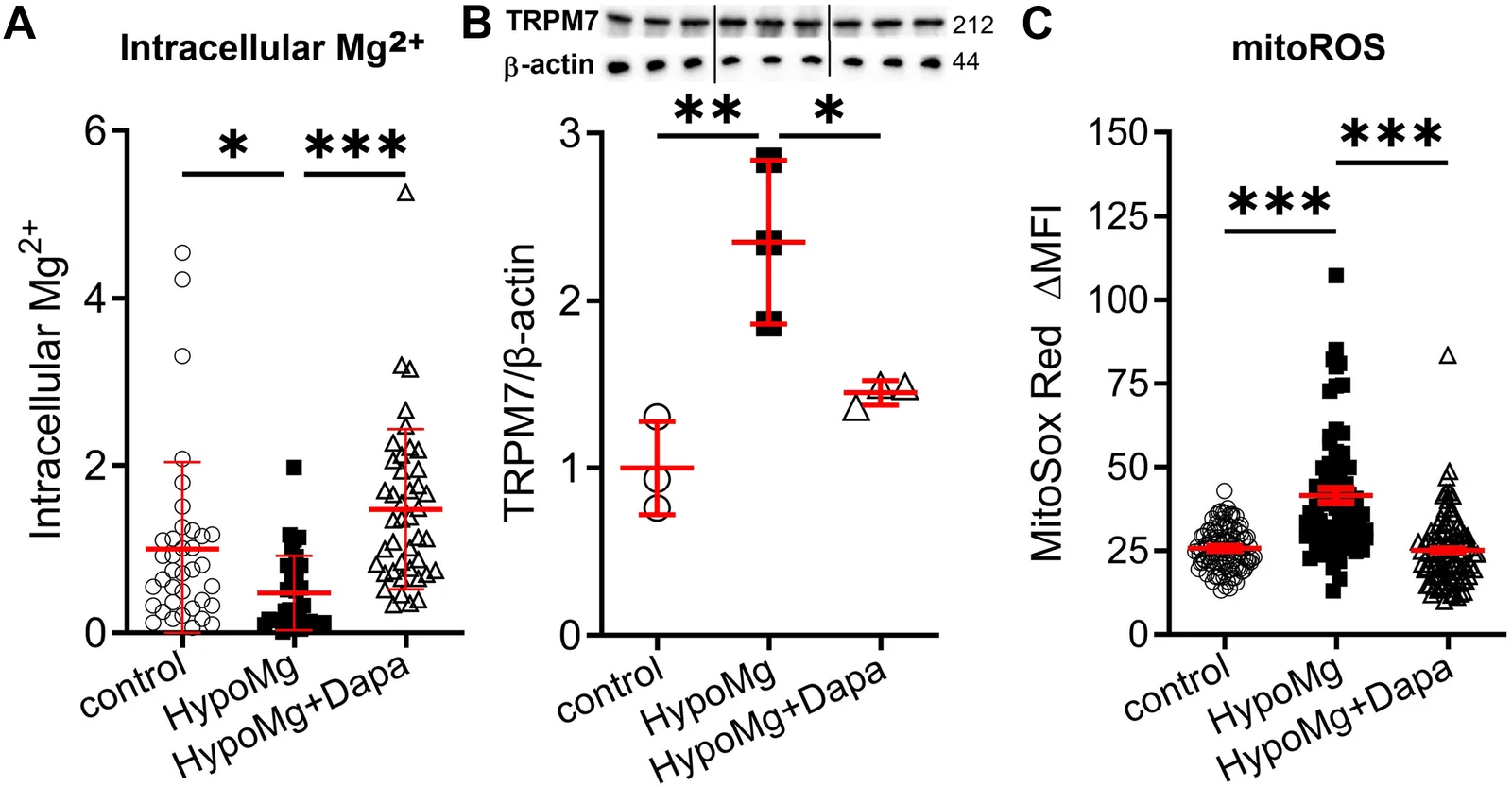
Dapa Prevented HypoMg-Induced Changes in Human Cardiomyocytes Human RL-14 cardiomyocytes were cultured in a normal (0.81 mmol/L Mg^2+^) or low-Mg (0.04 mmol/L Mg^2+^) media in the absence or presence of Dapa (5 μmol/L) for 48 hours. (A) Dapa treatment prevented HypoMg-induced reduction of intracellular Mg^2+^ measured with Mag-Fluo-4 AM fluorescence by confocal imaging; 35 to 42 cells from 3 dishes of cells/group were tested. (B) Dapa treatment suppressed HypoMg-induced TRPM7 overexpression in RL-14 cardiomyocytes with Western blot bands (protein molecular weight in kDa) shown on top; 3 dishes of cells/group were tested. (C) Dapa treatment suppressed HypoMg-induced mitoROS overproduction measured with MitoSox Red fluorescence; 72 to 112 cells from 3 dishes of cells/group were tested. Red bars in figures are mean ± SD. One-way analysis of variance with Tukey's multiple comparisons test was used for statistical analysis. ∗*P* < 0.05, ∗∗*P* < 0.01, ∗∗∗*P* < 0.001. Abbreviations as in Figures 1, 3, and 4.

### Dapa effects are analogous to inhibiting cardiomyocyte NHE1

The mechanism of actions for flozins in the kidney is binding the SGLT2, but studies show that SGLT2 is not expressed in the heart of humans or mice.^17^^,^^18^ We also could not detect either SGLT1 or SGLT2 messenger RNA and protein expression in the ventricles of control or HypoMg mice by RT-PCR (reaction cycle number >33) or mass spectrometry, respectively, confirming the Dapa effects were independent of SGLT2. NHE1 has been identified as a possible target of SGLT2 inhibitors.^60^, ^61^, ^62^, ^63^, ^64^, ^65^ In the mouse ventricles, we observed significant increases of NHE1 protein expression under HypoMg (Supplemental Figure 3).

NHE1 activation with the NHE1 activator rosmarinic acid (RA)^52^ mimicked the HypoMg effects such as increasing intracellular pH (pHrodo Green with higher fluorescent intensity indicating lower pH: 1,439 ± 381 for control; 1,241 ± 357 for RA, *P* = 0.011 vs control; 1,386 ± 332 for RA + Dapa, *P* = 0.99 vs control and *P* = 0.005 vs RA; 1,304 ± 362 for HypoMg, *P* = 0.015 vs control; Figures 6A and 6B) and elevating mitoROS levels (95 ± 38 for control; 128 ± 64 for RA, *P* < 0.001 vs control; 85 ± 33 for RA + Dapa, *P* = 0.77 vs control and *P* < 0.001 vs RA; 151 ± 75 for HypoMg, *P* < 0.001 vs control; Figures 6C and 6D), as well as decreasing intracellular free Mg^2+^ concentrations (Supplemental Figure 4). On the other hand, NHE1 inhibition with cariporide^51^^,^^60^^,^^65^ and Dapa had identical effects on preventing HypoMg-induced changes including a decreased intracellular pH (1,487 ± 240 for HypoMg + Dapa, *P* = 0.97 vs control and *P* = 0.003 vs HypoMg; 1,479 ± 494 for HypoMg + cariporide, *P* = 0.99 vs control, *P* = 0.005 vs HypoMg, and *P* > 0.99 vs HypoMg + Dapa; Figures 6A and 6B) and reduced mitoROS (100 ± 38 for HypoMg + Dapa, *P* = 0.98 vs control and *P* < 0.001 vs HypoMg; 101 ± 40 for HypoMg + cariporide, *P* = 0.97 vs control, *P* < 0.001 vs HypoMg, and *P* > 0.99 vs HypoMg + Dapa; Figures 6C and 6D). The Dapa effect was not explained by a reduction in NHE1 protein abundance, however (Supplemental Figure 3).

**Figure 6:**
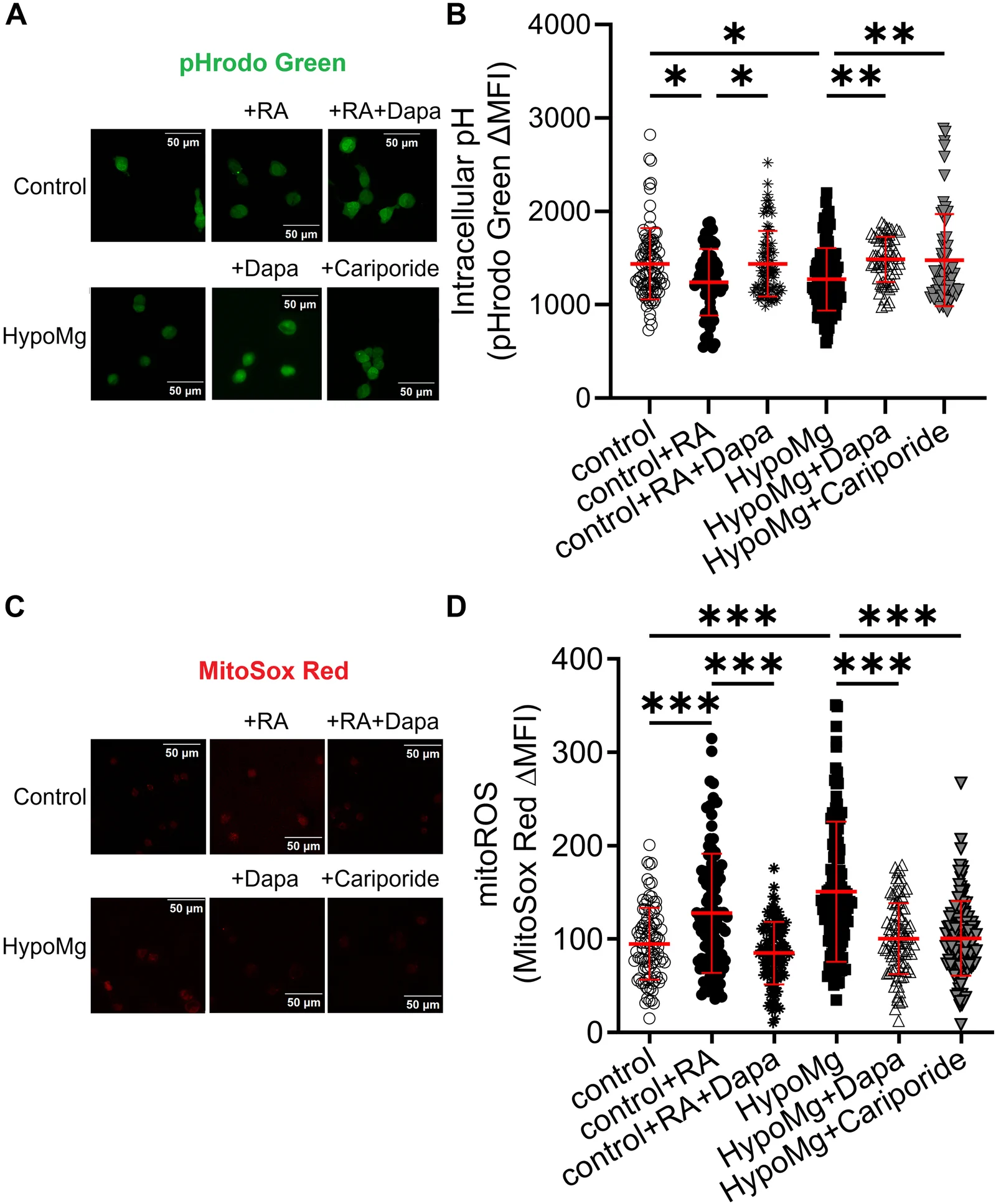
Dapa Inhibited NHE1 Activation or HypoMg Induced Changes in Cardiomyocytes Cell treatments were the same as in Figure 5; cariporide (10 μmol/L) or RA (10 μg/mL) was applied to cells for 48 hours. (A and B) Intracellular pH levels of RL-14 cardiomyocytes were measured with pHrodo Green fluorescence by confocal imaging. (A) Representative fluorescent images of pHrodo Green fluorescence. (B) ΔMFI is the fluorescent intensity difference of pHrodo Green between the cells and the background in the same image, higher fluorescence indicates low acidic pH; 62 to 125 cells from 3 dishes/group were tested. (C and D) mitoROS levels of RL-14 cardiomyocytes were measured with MitoSox Red fluorescence. (C) Representative fluorescent images of MitoSox Red. (D) ΔMFI is the fluorescent intensity difference of MitoSox Red between the cells and the background in the same image; 67 to 145 cells from 3 dishes/group were tested. Red bars in figures are mean ± SD. One-way analysis of variance with Tukey's multiple comparisons test was used for statistical analysis. ∗*P* < 0.05, ∗∗*P* < 0.01, ∗∗∗*P* < 0.001. RA = rosmarinic acid; other abbreviations as in Figures 1 and 3.

### NHE1 overexpression in cardiomyocytes mimicked HypoMg-induced changes

With NHE1 overexpression (NHE1OE) in RL-14 cardiomyocytes, intracellular pH measured with pHrodo Green (with higher fluorescent intensity indicating lower pH) was higher than control cells (*P* < 0.001 vs control) and returned to normal with Dapa treatment (*P* = 0.13 vs control and *P* < 0.001 vs NHE1OE; Figure 7A). Intracellular Mg^2+^ levels were decreased by NHE1OE (0.6 ± 0.2-fold of control, *P* = 0.004 vs control) and normalized by Dapa treatment (0.9 ± 0.4-fold of control, *P* = 0.94 vs control and *P* < 0.001 vs NHE1OE; Figure 7B). TRPM7 protein expression levels were elevated by NHE1OE (1.7 ± 0.2-fold of control for NHE1OE, *P* = 0.022 vs control) and normalized by Dapa treatment (1.1 ± 0.2-fold of control for NHE1OE + Dapa, *P* = 0.98 vs control and *P* = 0.021 vs NHE1OE; Figure 7C). NHE1OE was confirmed with 1.5 ± 0.3-fold elevation in the NHE1OE group (*P* = 0.040 vs control; Figure 7D). Interestingly, NHE1OE was suppressed by Dapa treatment (0.7 ± 0.2-fold of control for the NHE1OE + Dapa group, *P* = 0.35 vs control and *P* = 0.004 vs NHE1OE). As shown in Figure 7E, mitoROS levels were increased in NHE1OE cells (*P* < 0.001 vs control) and reduced with NHE1OE + Dapa (*P* < 0.001 vs control and *P* < 0.001 vs NHE1OE). When TRPM7 kinase inhibitor TG100-115^66^^,^^67^ (10 mmol/L final concentration) was applied to the NHE1 overexpressed cells, mitoROS levels were normalized (135 ± 59 for control; 372 ± 88 for NHE1OE, *P* < 0.001 vs control; and 122 ± 37 for NHE1OE + TG100-115, *P* = 0.42 vs control and *P* < 0.001 vs NHE1OE). This suggests that NHE1 modulated mitoROS via TRPM7 kinase.

**Figure 7:**
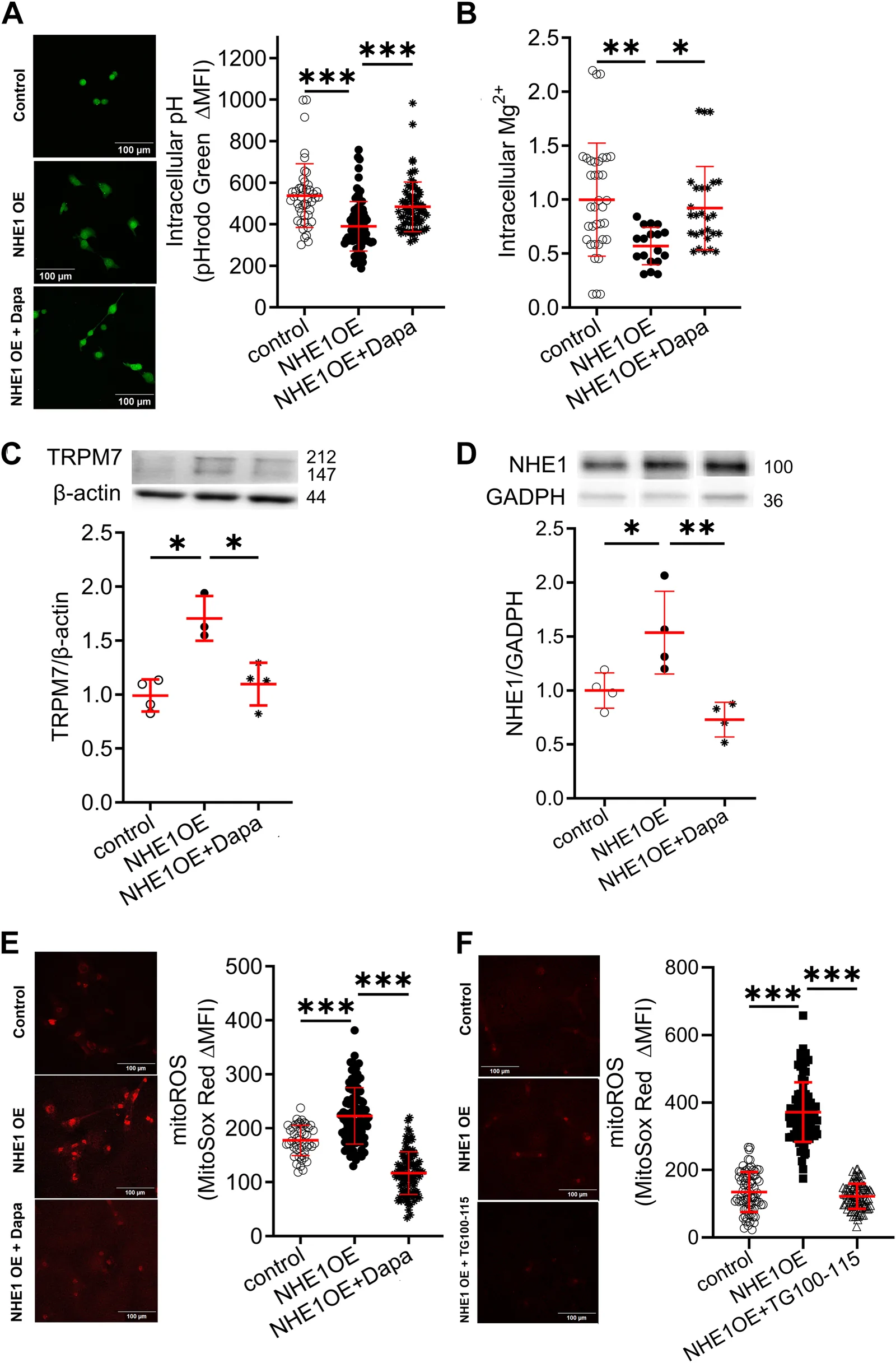
Dapa Inhibited Changes Mediated by NHE1 Overexpression HypoMg and Dapa treatment were the same as in Figure 5; NHE1 overexpression was achieved as described in the methods section. (A) Intracellular pH levels are shown with representative images of pHrodo Green fluorescence (left) and ΔMFI values (right) with the same methods as stated in Figure 5; 45 to 83 cells from 3 dishes/group were tested. (B) Intracellular Mg^2+^ was measured with Mag-Fluo-4 AM fluorescence; 18 to 35 cells from 3 dishes/group were tested. (C and D) Protein levels of TRPM7 and NHE1 were measured with stand Western blot and representative Western blot bands are shown with protein molecular weight in kDa; 3 to 4 dishes of cells/group were measured. (E) mitoROS levels of RL-14 cardiomyocytes of RL-14 cardiomyocytes with NHE1 overexpression and Dapa treatment are shown with representative images of MitoSox Red fluorescence (left) and ΔMFI values (right); 47 to 99 cells from 3 dishes/group were tested. (F) mitoROS levels of of RL-14 cardiomyocytes with NHE1 overexpression and TG100-115 treatment (10 mmol/L final concentration) are shown with representative images of MitoSox Red fluorescence (left) and ΔMFI values (right); 76 to 99 cells from 3 dishes/group were tested. Scale bars in (A), (E), and (F) indicate 100 μm. Red bars in figures are mean ± SD. One-way analysis of variance with Tukey's multiple comparisons test was used for statistical analysis. ∗*P* < 0.05, ∗∗*P* < 0.01, ∗∗∗*P* < 0.001. NHE1 = sodium-hydrogen exchanger 1; NHE1OE = sodium-hydrogen exchanger 1 overexpression; other abbreviations as in Figures 1 and 3.

## Discussion

Flozin class SGLT2 inhibitors such as dapagliflozin and empagliflozin improve symptoms and reduce hospitalizations in patients with HFpEF^12^^,^^13^ and patients with HFrEF^68^ but the mechanism of this effect is unknown with SGLT2 mainly expressed in the kidney, with no expression in the heart.^17^^,^^18^ In this study, we found that Dapa improved HFpEF, reduced cardiac inflammation, and decreased cardiomyocyte oxidative stress through modulation of NHE1 activity.

There is some controversy about whether SGLT2 inhibitors can block NHE1.^69^^,^^70^ Although we did not measure NHE1 activity directly as sodium-dependent pH changes after acid challenge, we show that Dapa reversed the intracellular alkalinization mediated by NHE1 activation, acted in an identical manner as a known NHE1 inhibitor (Cariporide) to reverse the alkalinization induced by HypoMg, and blocked the alkalinization caused by NHE1 overexpression. Together, these data indirectly suggest that Dapa inhibits cardiac NHE1. In vivo, levels of NHE1 were increased with DD, supporting the idea that NHE1 may play a role in HFpEF. Furthermore, it is unlikely that Dapa had its effect through SGLT2 inhibition, because cardiac SGLT2 is absent in our model.

Our previous studies have shown HypoMg plays important roles in the pathology of diabetes-induced HFpEF and that Mg^2+^ can act as an antioxidant by downregulating the Mg^2+^ transporter TRMP7, whose kinase function phosphorylates complex II of the mitochondrial electron transport change to cause excess mitoROS.^21^^,^^28^ Excess cardiomyocyte oxidative stress results in S-glutathionylation of cMyBPC that can explain the DD.^21^^,^^54^^,^^55^ The role of Mg^2+^ in human HF is controversial, however.^71^^,^^72^ It is clear that diabetic patients have lower serum Mg^2+^,^71^, ^72^, ^73^ and recently we have shown that low Mg^2+^ can cause HFpEF and that Mg^2+^ repletion can reverse the diabetes mellitus (DM)-associated DD.^21^^,^^22^^,^^28^ Here, we used the HypoMg model as a reasonable phenocopy of diabetic DD that is insensitive to changes in glucose levels. Nevertheless, because there is no universally agreed model of human HFpEF, care should be used when extrapolating these results to diabetic HFpEF models, other HFpEF models, or human disease.

In our study, Dapa corrected the serum HypoMg, but in human studies, the effect of SGLT2i on serum Mg^2+^ is much less dramatic despite their improvement in cardiovascular outcomes.^71^, ^72^, ^73^ Although all of the mechanisms by which Dapa affected serum Mg^2+^ in our study are unclear, the combined human and mouse data suggest that the relevant Dapa effect may be on intracellular Mg^2+^, not serum Mg^2+^. NHE1 activation is known to cause intracellular HypoMg,^74^^,^^75^ albeit the mechanism is unclear. In our experiments, Dapa inhibited NHE1 activity, raised intracellular Mg^2+^ levels, decreased TRPM7 levels presumably by affecting Mg^2+^-dependent transcription,^76^ and improved mitochondrial dysfunction (Figure 8), suggesting that the effect of Dapa on intracellular Mg^2+^ was through NHE1 inhibition and that the NHE1 effect was mediated by intracellular Mg^2+^-dependent TRPM7 regulation. Moreover, these data suggest that increases in Mg^2+^ transport of TRPM7 are not responsible for the effects of Dapa, because Mg^2+^ transport would be expected to be reduced when TRPM7 levels are lower with Dapa.

**Figure 8:**
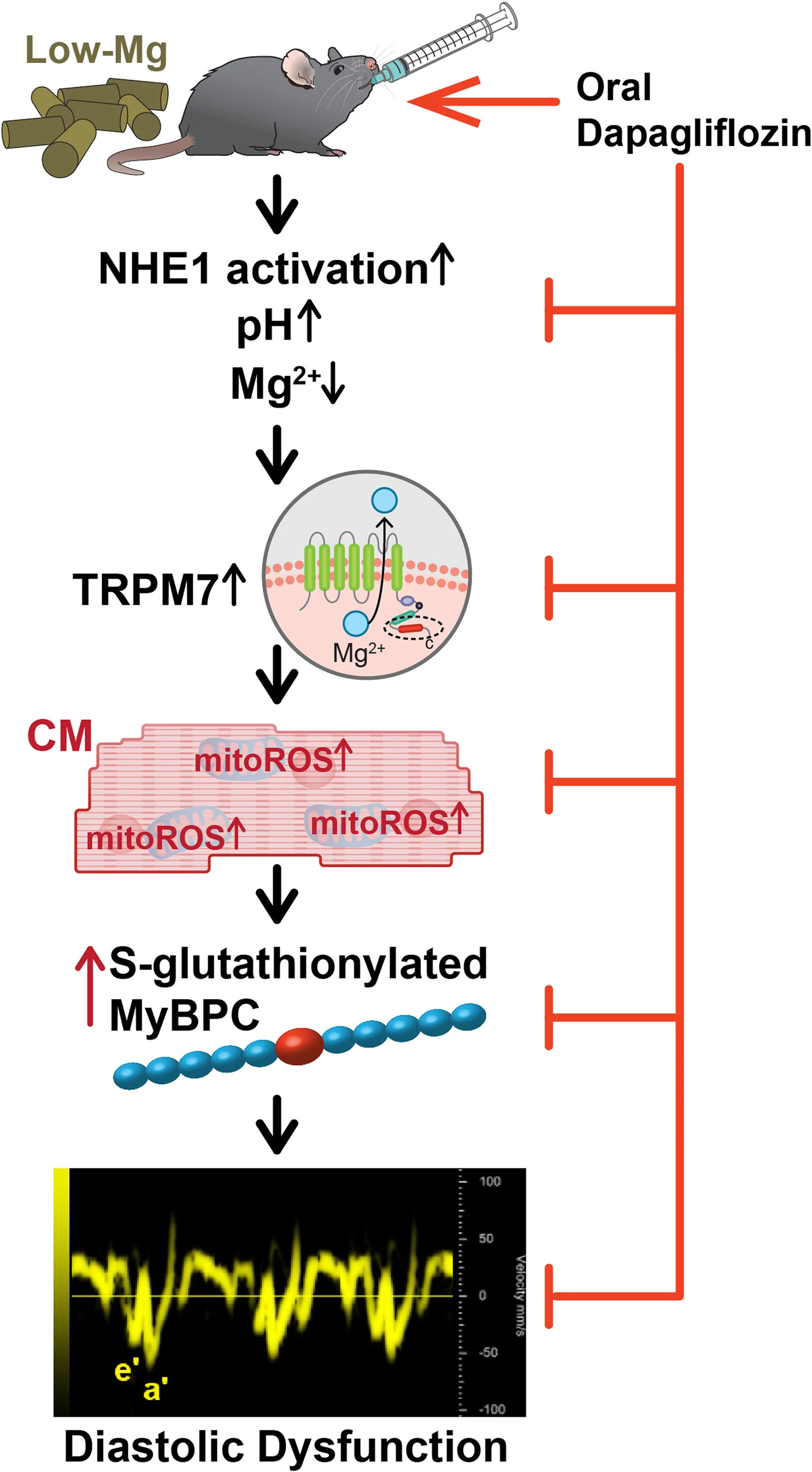
The Mechanism of Dapa Protection Against Cardiac Diastolic Dysfunction and Heart Failure With Preserved Ejection Fraction HypoMg mice had activated NHE1, increased intracellular pH, decreased serum and intracellular Mg^2+^, TRPM7 overexpression, cardiomyocyte oxidative stress with increased mitoROS production, elevated cMyBPC *S*-glutathionylation, and cardiac diastolic dysfunction. Dapa treatment improved cardiac diastolic function by inhibiting NHE1 activity, decreasing intracellular pH, increasing serum and intracellular Mg^2+^, suppressing TRPM7 overexpression, and reducing mitochondrial oxidative stress. Abbreviations as in Figures 1, 3, and 7.

Just like systolic HF is a syndrome with risk factors, proximal causes, cardiac contractile dysfunction, and systemic sequelae, diastolic HF is also a syndrome. In each case, however, the focus is on treating the cardiac dysfunction to resolve the systemic sequelae. Previously, we have shown that the HypoMg model is a reasonable experimental phenocopy of DM-induced HFpEF that is insensitive to any Dapa-mediated changes in glucose, and both models show DD at the myofilament, myocyte, whole heart, and animal levels associated with an elevated LVEDP, the sine qua non of HF, and normalization in the LVEDP is associated with echocardiographic and molecular marker improvements.^21^^,^^22^^,^^28^ Here, we show that Dapa treatment also improves the echocardiographic and molecular markers of DD in a similar manner.

Dapa seems to have its effect on lusitropy through NHE1, which is a structurally related to SGLT2. NHE1 overexpression or activation mimicked HypoMg-induced changes on intracellular pH, intracellular Mg^2+^, TRPM7 expression, and cardiomyocyte mitoROS, and Dapa suppressed these changes, indicating that Dapa plays its roles through modulating NHE1 activity. In addition, TRPM7 kinase inhibition prevented NHE1 overexpression-induced mitoROS overproduction, suggesting that NHE1 modulates mitoROS through TRPM7 kinase. Although we did not investigate whether Dapa reversed all systemic sequalae of HFpEF, Dapa has demonstrated the ability to reduce overall complications in human HFpEF,^13^^,^^77^^,^^78^ suggesting it is likely to have the same effect in our experiments.

The antioxidant effects of Dapa are consistent with previous literature. For example, Dapa suppresses oxidative stress and improves mitochondrial function in mouse heart,^79^ human skeletal muscle fibers,^80^ and human HK-2 cells.^50^ In diabetic mouse kidneys, Dapa shows protection against oxidative stress with reduced NOX4 levels, similar to our observation with HypoMg mouse hearts.^45^ Empagliflozin improves the activities of mitochondrial electron transport chain in rat DD and HFpEF.^81^ Empagliflozin also decreases mitochondrial oxidative stress in mouse myocardial ischemia reperfusion injury,^82^ improves mitochondrial function and attenuates mitoROS in diabetic cardiomyopathy,^40^ and reduces oxidative stress in rat failing hearts.^83^ Canagliflozin boosts cardiomyocyte mitochondrial homeostasis with decreased NOX4 and oxidative stress and attenuated inflammation in mouse and rat hearts.^84^, ^85^, ^86^ SGLT2 inhibitors interact with NHE1 and decrease NHE1 activity.^62^^,^^65^^,^^87^ NHE1 is expressed in cardiomyocytes,^88^^,^^89^ and our studies with RL-14 cardiomyocytes showed that NHE1 overexpression or activation can mimic HypoMg-induced intracellular pH elevation, intracellular Mg^2+^ reduction, TRPM7 overexpression, and mitoROS overproduction, whereas NHE1 inhibition can mimic Dapa's effect to prevent these HypoMg-induced changes. These observations imply that Dapa likely plays its protective effect by inhibiting cardiac NHE1.

NHE1 inhibition has been tested clinically in several trials. Both the Guard During Ischemia Against Necrosis (GUARDIAN) and sodium-hydrogen EXchange inhibition to Prevent coronary Events in acute cardiac conDITIONs (EXPEDITION) trials suggested a cardiac benefit to NHE1 inhibition with cariporide during cardiac bypass surgery.^90^ In the EXPEDITION trial, an increase in cerebral events was noted, however. A different NHE1 inhibitor, eniporide, gave no benefit when administered before reperfusion therapy in acute myocardial infarction (ESCAMI [Evaluation of the Safety and Cardioprotective Effects of Eniporide in Acute Myocardial Infarction] trial).^91^ On the other hand, SGLT2 inhibition has shown clear clinical benefits in HFpEF and HFrEF.^12^^,^^13^^,^^68^^,^^77^^,^^78^ There are many possible differences that could explain the variable outcomes of NHE1 inhibition with SGLT2 inhibitors and more traditional NHE1 blockers. These differences may include the diseases treated, the duration of treatment (acute vs chronic), the efficacy and side-effect profile of the drugs, or additional benefits of SGLT2i over just NHE1 inhibition.

Our previous studies have shown that cardiac inflammation and macrophage activation are necessary for diabetic and HypoMg-induced DD and HFpEF.^21^^,^^22^^,^^24^^,^^25^^,^^28^ Here, we reported that Dapa can improve HypoMg-induced myocardial inflammation as evidenced by a shift of macrophage toward a more inflammation-resolving phenotype. Although further macrophage phenotyping will be important for a more refined understanding of the cardiac immunology, we show that Dapa decreased total cardiac macrophage cell infiltration, IL-1β, and IL-18, demonstrating that the overall effect of Dapa was anti-inflammatory. Because most cardiac IL-1β present in heart during HFpEF is secreted by macrophages,^24^ it is likely the balance of proinflammatory and proresolving macrophages was altered by Dapa. This effect of Dapa is consistent with previous literature (for review see Luna-Marco et al^41^ and Li et al^42^). NHE1 is expressed in macrophages, but it remains to be seen if the anti-inflammatory effect of Dapa is secondary to NHE1 inhibition in this cell type.

### Study limitations

One limitation of these results is that only male HypoMg mice were studied because of excess mortality in the females exposed to HypoMg.^22^ The control and HypoMg-Dapa groups used both male and female mice, however. Previously, we have shown that this mortality is related to more severe brain mitochondrial dysfunction and subsequent seizure activity in females.^56^ Therefore, it seems likely that our findings would apply to female mice as well and the amplfied response may help explain the increased proclivity for HFpEF in female humans.^92^^,^^93^ When compared with human disease, the HypoMg-induced DD model has a lower serum Mg^2+^ level than is common in humans with DM, so the Dapa effects may be more pronounced. Finally, the data cannot exclude a role of pH changes in the lusitropic effect of Dapa.

## Conclusions

SGLT2 inhibitor Dapa prevented HypoMg-mediated HFpEF by reducing myocardial inflammation and cardiomyocyte mitochondrial dysfunction. The Dapa-mediated reduction in cardiomyocyte oxidative stress appeared to be the result of NHE1 inhibition that caused lower intracellular pH, increased intracellular Mg^2+^ levels, and decreased TRPM7 protein expression. These results explain the efficacy of Dapa and suggest that NHE1 inhibition may represent a new target for treatment of HFpEF.

## Funding Support and Author Disclosures

This study was supported by the following grants to Dr Dudley: R01 HL165704, R01 HL177974, and R56 HL162208. All other authors have reported that they have no relationships relevant to the contents of this paper to disclose.

Perspectives **COMPETENCY IN MEDICAL KNOWLEDGE:** SGLT2 inhibitors are indicated for the treatment of human HFpEF. With no SGLT2 present in cardiomyocytes, SGLT2 inhibitors act on NHE1 to raise intracellular Mg^2+^, reduce TRPM7, and decrease mitoROS production, leading to improved relaxation. SGLT2 inhibitors also enhance the percentage of anti-inflammatory macrophages in the heart.

**TRANSLATIONAL OUTLOOK 1:** Sodium-hydrogen exchange inhibition may be a novel target for the prevention and treatment of HFpEF.

## Appendix

Supplemental Material 2

Supplemental Supporting Data Values
