# Supplementary material for: Dapagliflozin Prevents Heart Failure With Preserved Ejection Fraction by Inhibiting the Sodium/Hydrogen Exchanger
Source: JACC Basic Transl Sci. 2026 Sep 18;11(10):101698. doi: 10.1016/j.jacbts.2026.101698 (PMC13626939; doi:10.1016/j.jacbts.2026.101698)

Complete unedited blot for all figures:

Figure 1B. Red rectangular region is shown in Figure 1B.

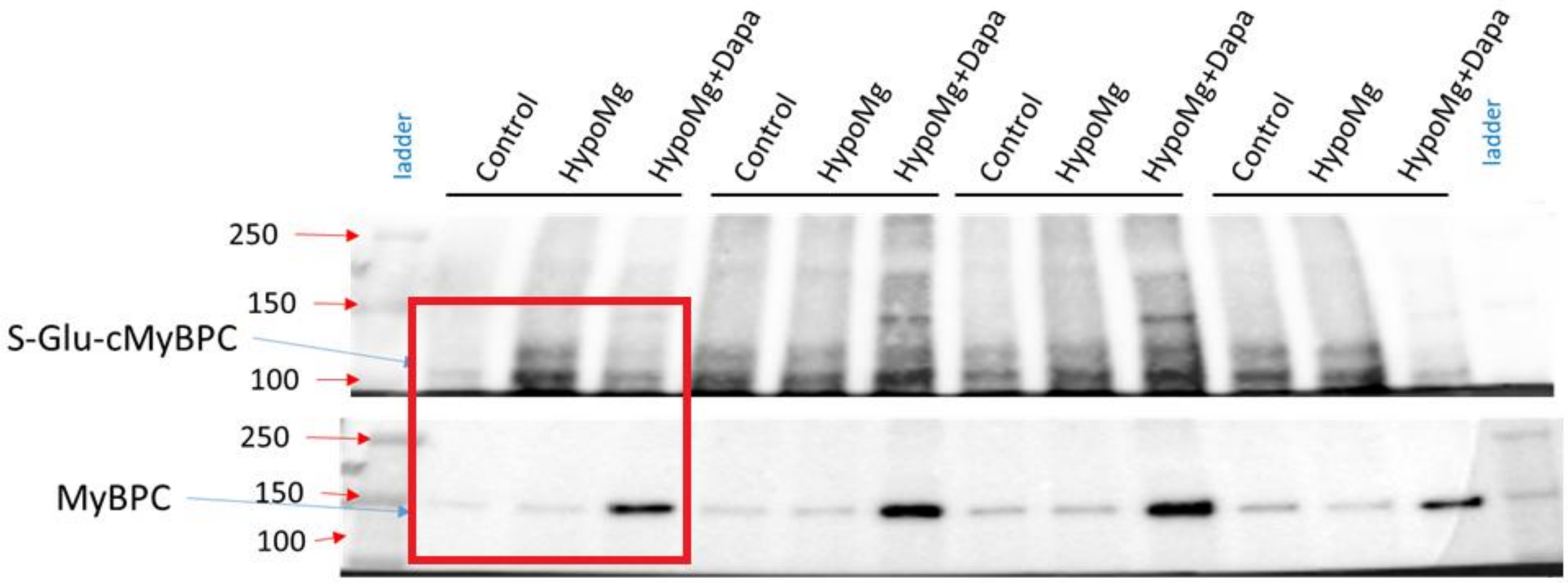

Figure 1C. Red rectangular region is shown in Figure 1C.

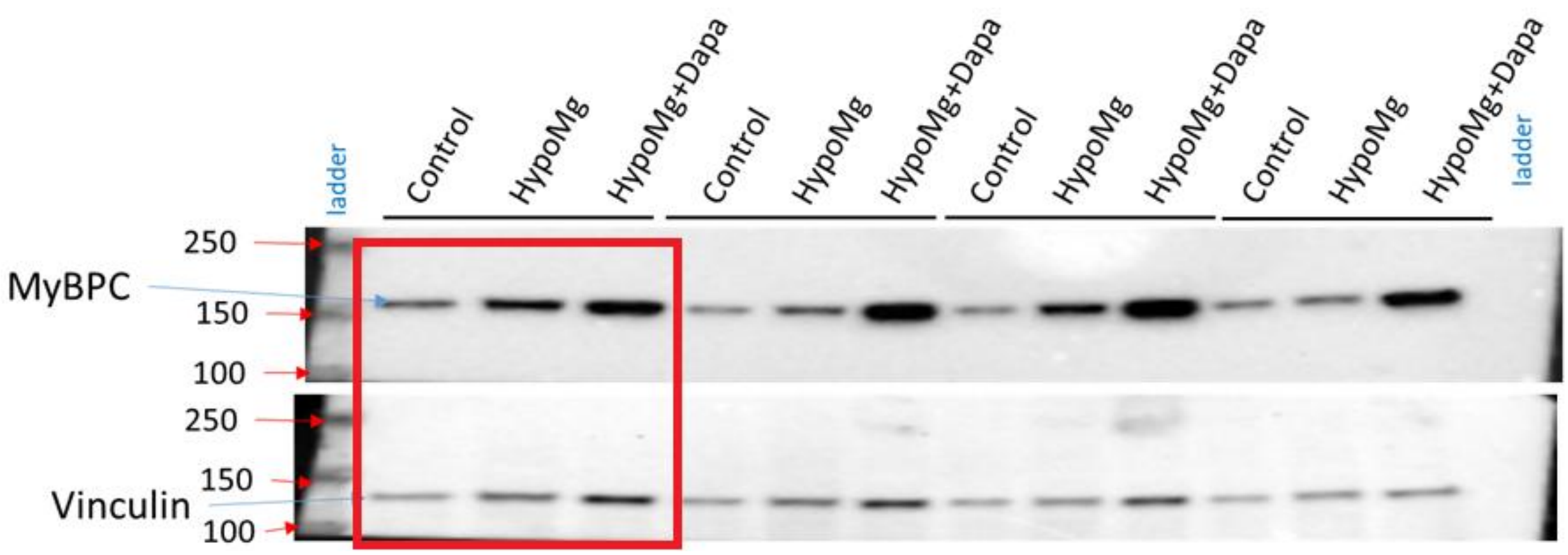

Figure 3A. Red rectangular region is shown in Figure 3A.

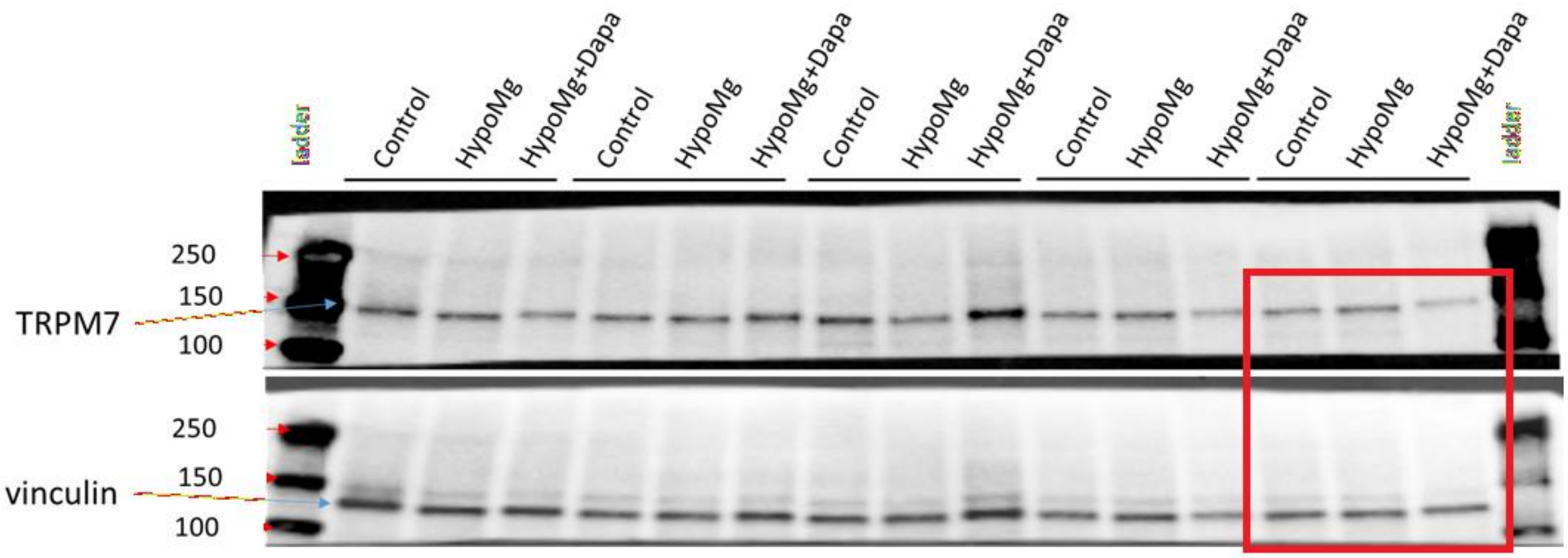

Figure 3C. Red rectangular region is shown in Figure 3C.

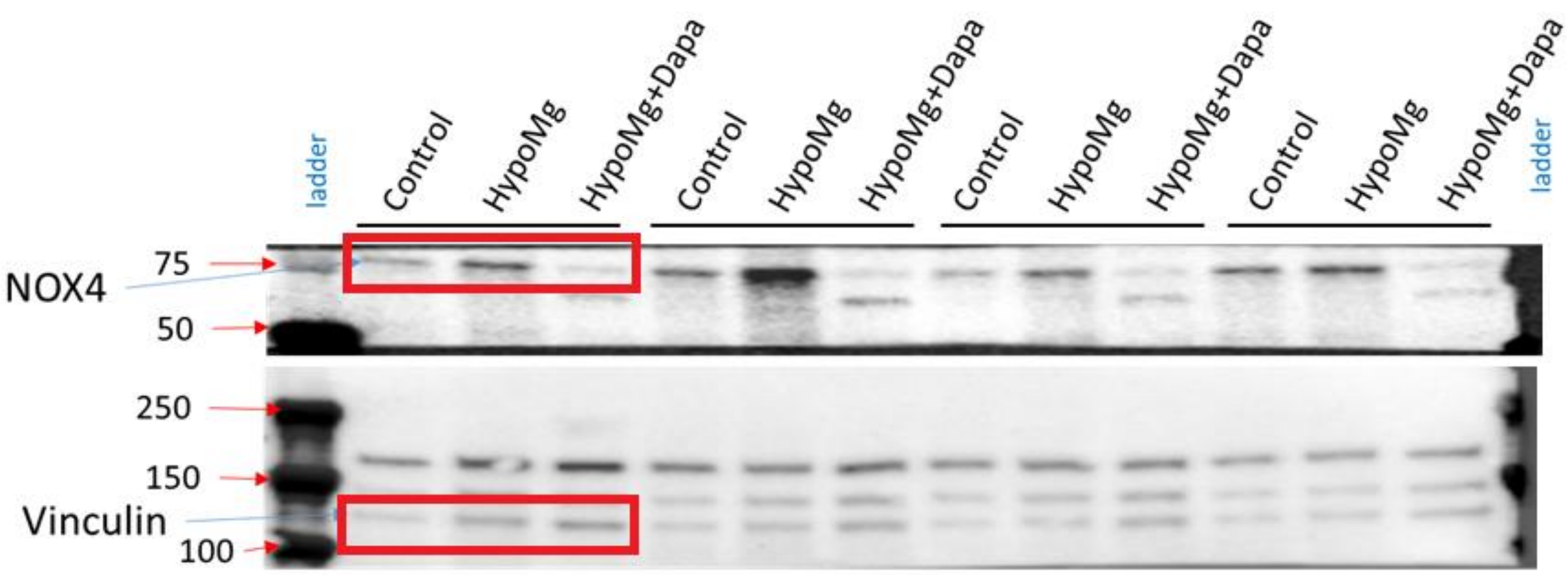

Figure 4F. Red rectangular region is shown in Figure 4F.

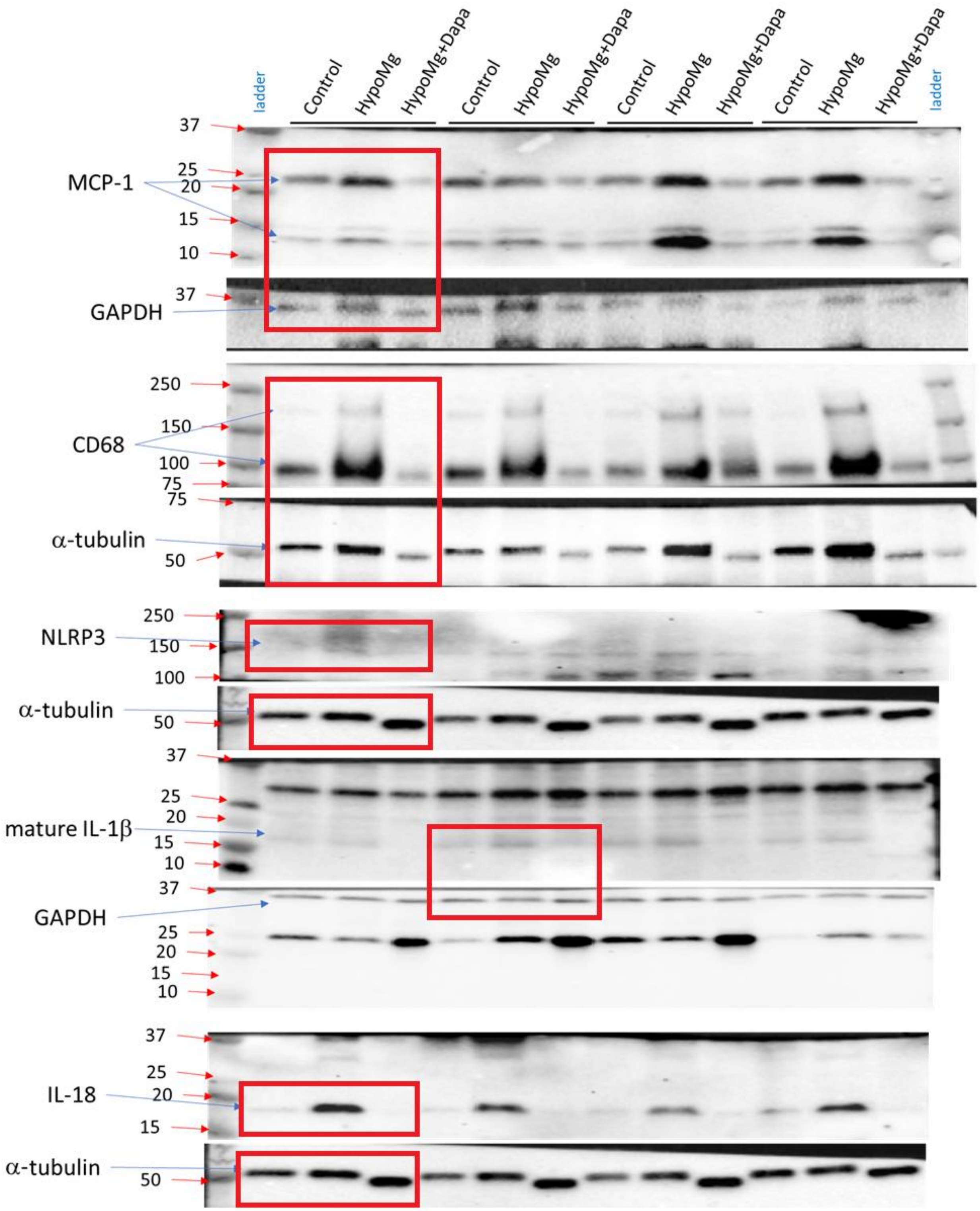

Figure 5B. The whole region is shown in Figure 5B.

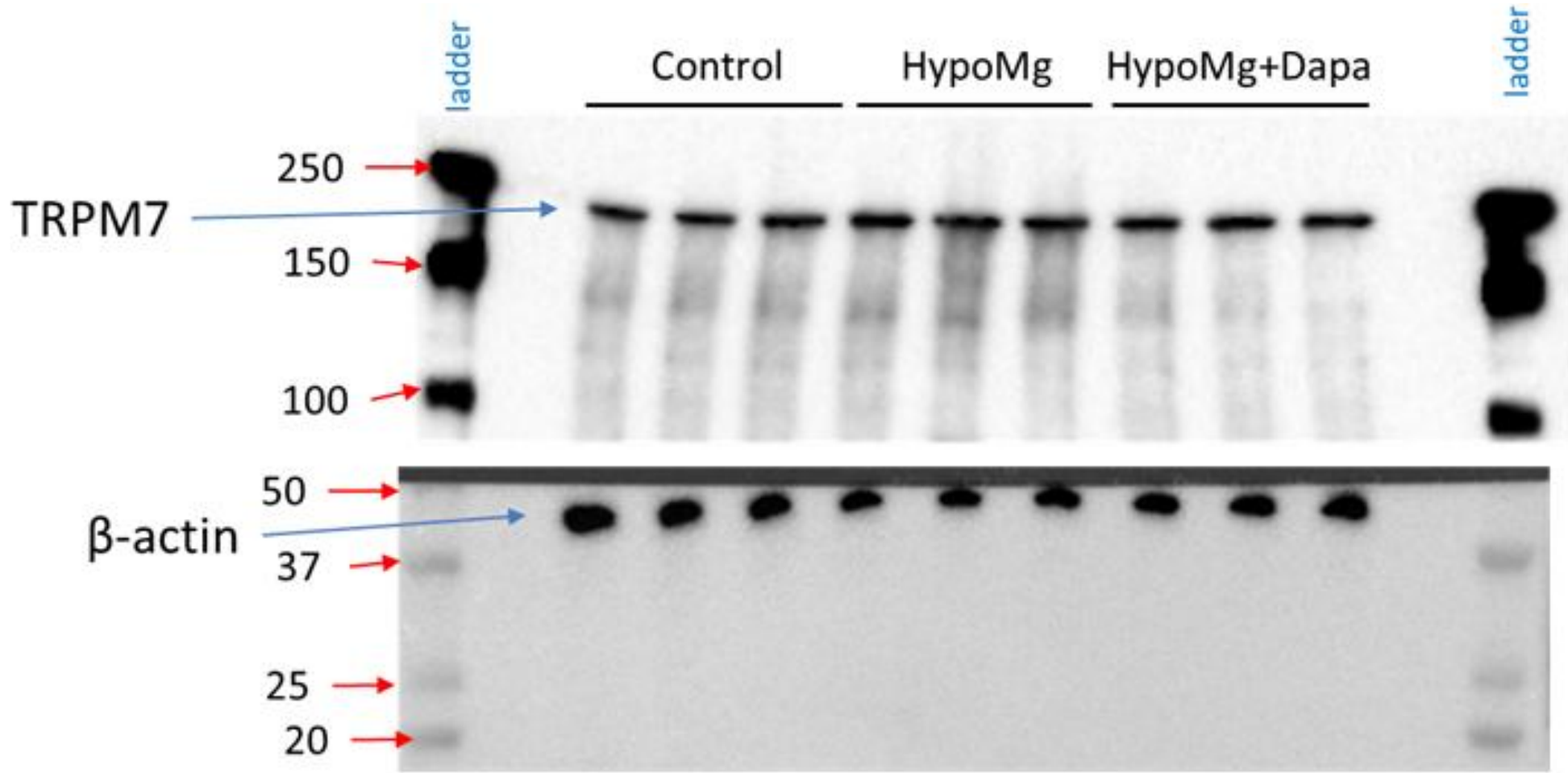

Figure 7D. Red rectangular region is shown in Figure 7D. The other three orange region, together with the red rectangular region were used for data analysis.

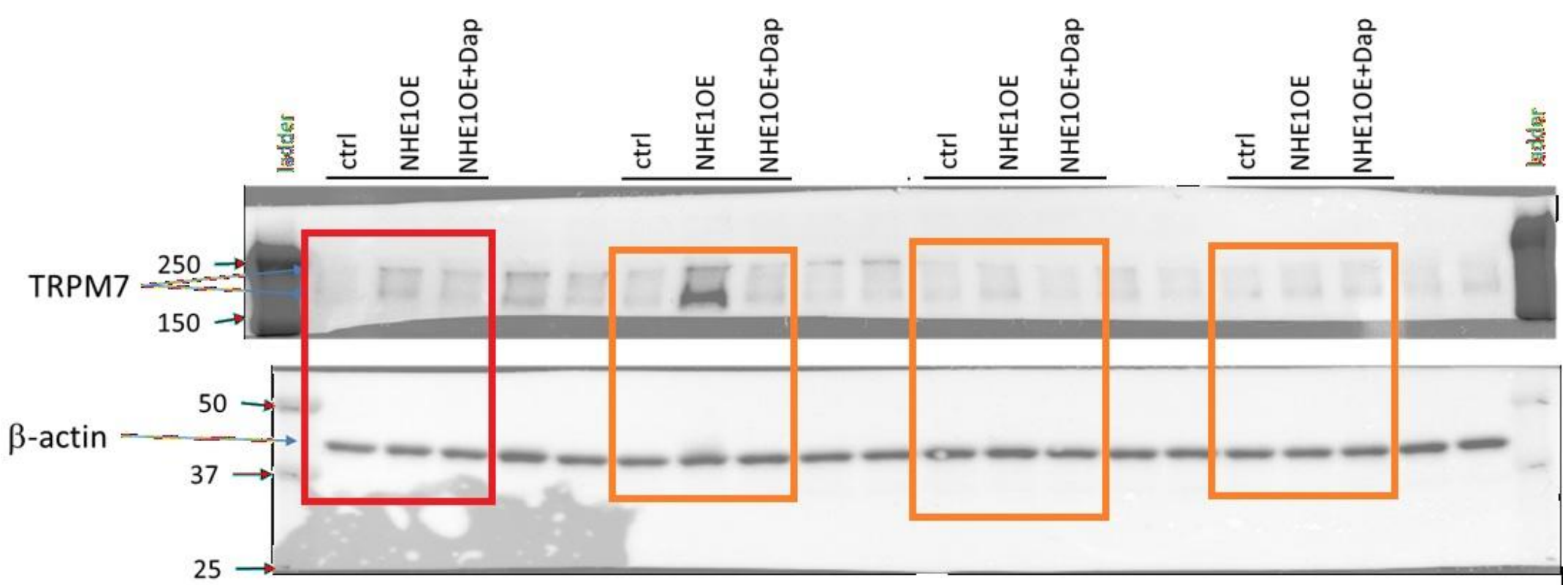

Figure 7E. Red rectangular region is shown in Figure 7E.

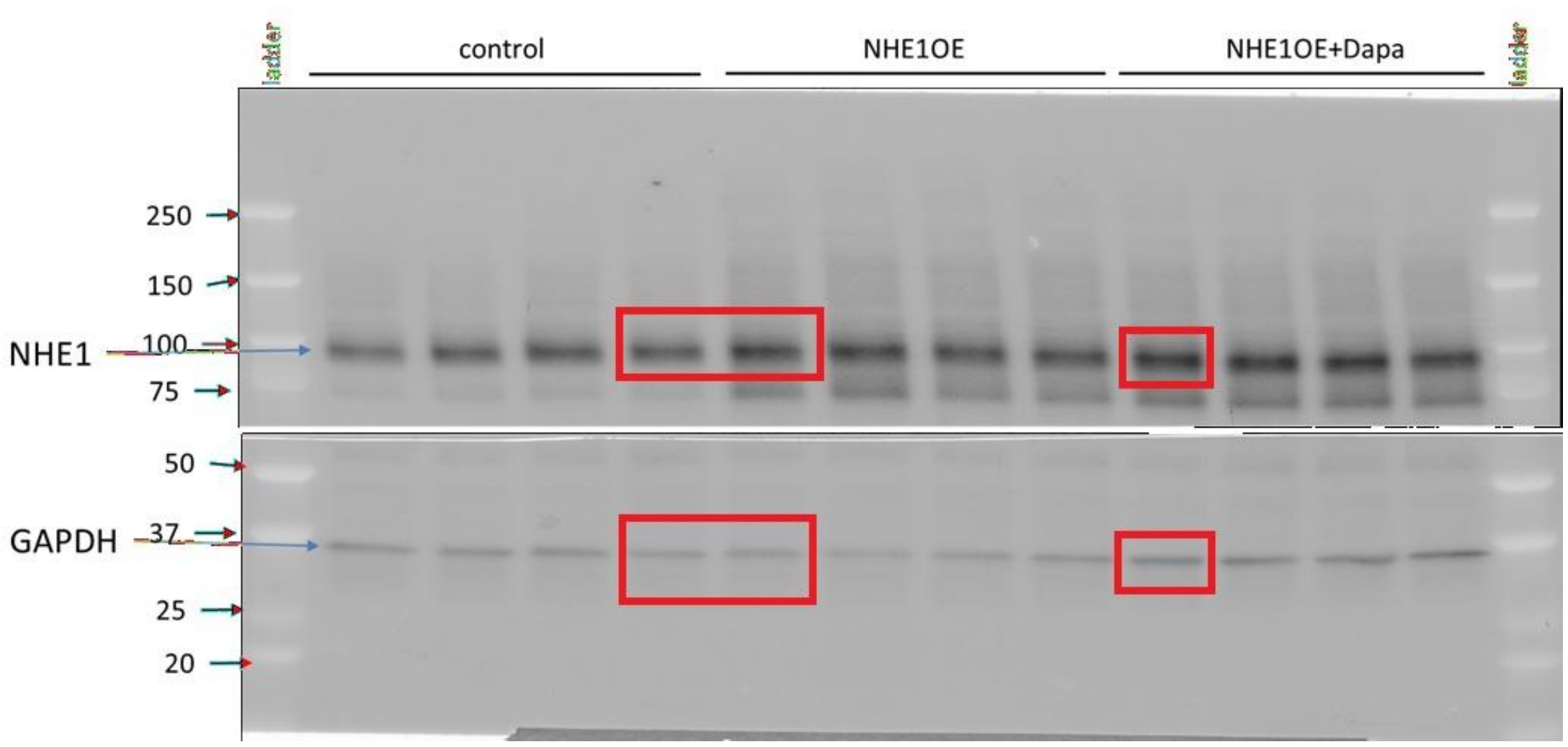

Supplementary Figure 2: The whole region is shown in Supplementary Figure 2

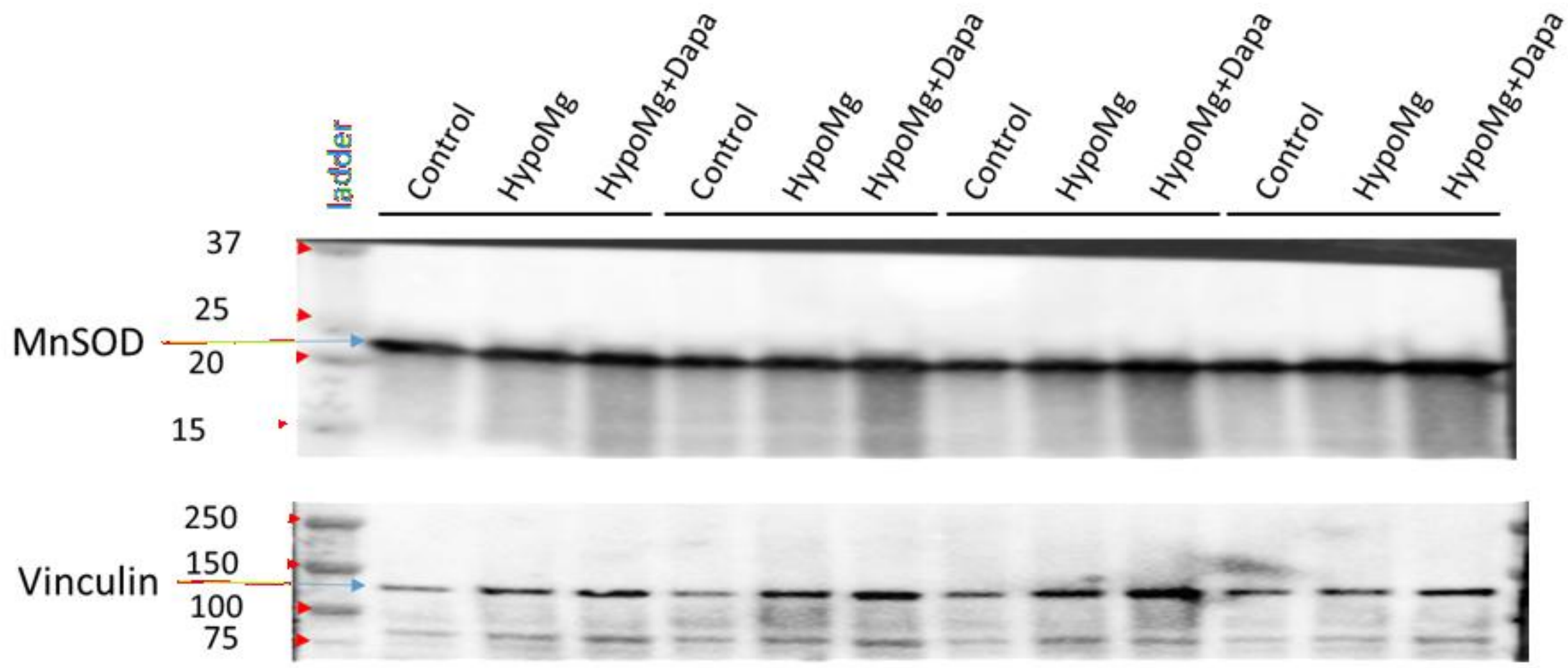

Supplementary Figure 3: Red rectangular region is shown in Supplementary Figure 3.

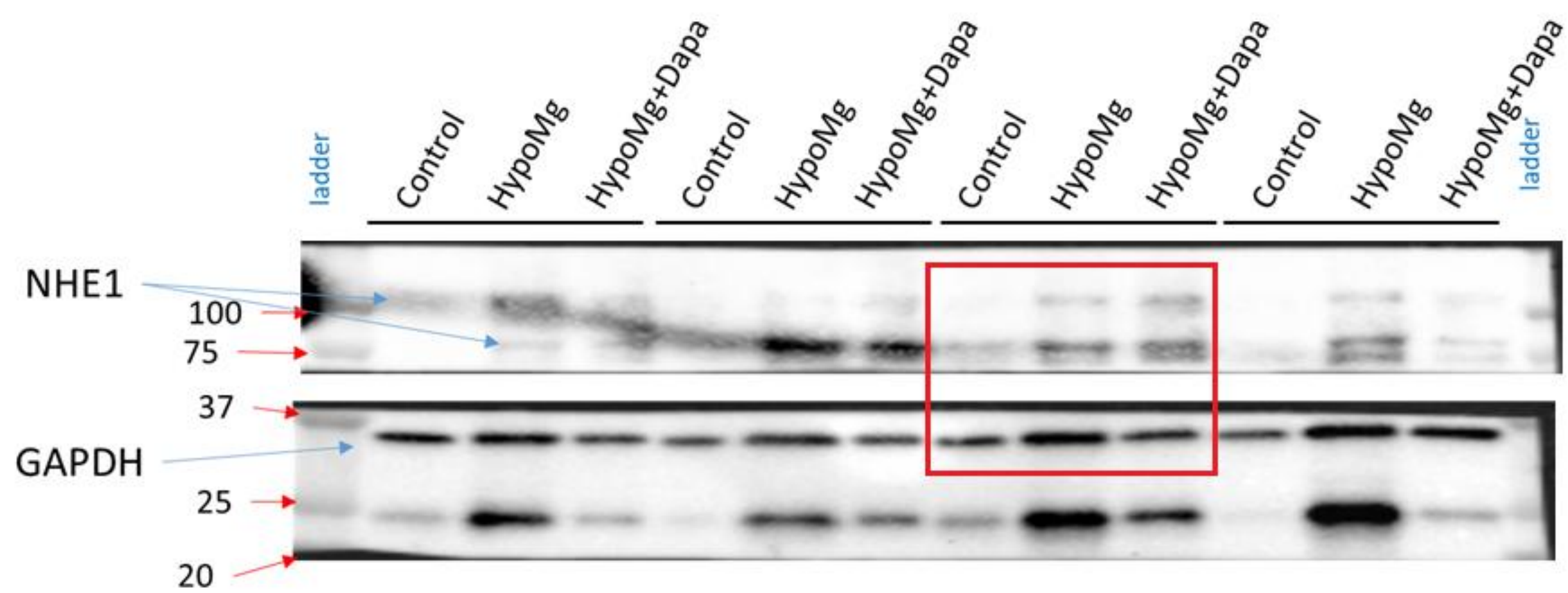

Supplement: Supplementary file 1 [file mmc1.pdf]
