# Supplementary material for: Dapagliflozin Prevents Heart Failure With Preserved Ejection Fraction by Inhibiting the Sodium/Hydrogen Exchanger
Source: JACC Basic Transl Sci. 2026 Sep 18;11(10):101698. doi: 10.1016/j.jacbts.2026.101698 (PMC13626939; doi:10.1016/j.jacbts.2026.101698)
Supplement: Supplementary file 2 [file mmc2.docx]

**Dapagliflozin prevents diabetic heart failure with preserved ejection fraction by inhibiting the sodium/hydrogen exchanger**

Man Liu, Hong Liu, Eunji Kim, Gyeoung-Jin Kang, Mitchell C. Neumann, Madeline Johnson, Ruthvika Murikinati, Samuel C. Dudley Jr*

Cardiovascular Division, Department of Medicine, the Lillehei Heart Institute, University of Minnesota at Twin Cities, Minneapolis, MN

***Address correspondence to:**

Dr. Samuel C. Dudley, Jr.

Professor of Medicine

Cardiovascular Division

Department of Medicine

Lillehei Heart Institute

University of Minnesota

CCRB 4-141

2231 6th Street SE

Minneapolis, MN 55455

Office: [612.624.8970](tel:(612)%20624-8970)

Fax: [612.626.4411](tel:(612)%20626-4411)

Email: [sdudley@umn.edu](mailto:sdudley@umn.edu)

**Methods**

Any supporting data not available within the article are available from the corresponding author upon reasonable request. Chemicals and reagents were purchased from Sigma-Aldrich (St. Louis, MO), except stated otherwise.

Sex as a biological variable*.* Both sexes of mice were studied in control and HypoMg+Dapa group, while mainly male mice were tested in HypoMg group because of excess mortality in the female mice exposed to HypoMg.^1^

Animal and cell treatment. C57BL/6J mice (The Jackson Laboratory, Strain #000664) were purchased from Jackson Laboratory (Bar Harbor, ME). Mice were randomly assigned to different groups at 10 weeks of age: (i) control mice fed with a normal chow (containing 2 g/kg Mg, Teklad global 18% protein rodent diet 2018, Envigo Teklad Diets, Madison, WI) for 6 weeks; (ii) HypoMg mice fed with a low-Mg diet (TD.93106, containing ~15-30 mg/kg Mg, Envigo Teklad Diets) for 6 weeks; (iii) HypoMg+Dapa mice fed with the low-Mg diet (6 weeks) and Dapa oral treatment (1.5 mg/kg/day, starting from the 4^th^ week of low-Mg diet and lasting for 3 weeks). Distilled and deionized H_2_O was given to the mice to control any possible Mg intake from drinking water. The low-Mg diet was chosen based on previous animal studies.^1-4^ The dose, treatment timing, and length of Dapa were modified from other animal studies with Dapa.^5,6^ The exact numbers of mice used in each experiment are shown in figure legends. The concentrations used in the cell model for Dapa and rosmarinic acidwere based on previous reports.^7-10^

Echocardiographic assessment. Noninvasive echocardiography was performed with a Vevo 2100 ultrasound system (VisualSonics, Toronto, Canada) as we have done previously.^1,11^ During the image acquisition, mice were anesthetized with 1-3% isoflurane and a heart rate ~400 beats/min was maintained. M-mode images in the parasternal long axis and the left ventricular (LV) short-axis views at the mid-papillary level were taken. The ratio between mitral peak early filling velocity (E) and longitudinal tissue velocity of the mitral anterior annulus (e’), E/e’, was assessed in the subcostal four-chamber view by pulse-wave and tissue Doppler imaging to evaluate diastolic relaxation function.^12^ . The absolute value of E/e’, following the usual convention, was used. Measurements were averaged from five consecutive beats during expiration. The images for each mouse were recorded for at least 5 s (30–40 cardiac cycles). For the baseline evaluation, the following LV diastolic and systolic dimensions were measured: the posterior wall thickness in diastole and in systole, the anterior wall thickness in diastole and in systole, and the LV end diastolic (LVEDD) and end systolic dimensions (LVESD). From these dimensions, the main functional parameter, percent LV ejection fraction (EF%) was calculated as 100 × (7/2.4 + LVEDD) × LVEDD^3^ – (7/2.4+LVESD) × LVESD^3^ / (7/2.4+LVEDD) × LVEDD^3^.

Western blotting for protein levels. Mouse ventricles of four mice from each group was collected and processed for Western blotting. For *S*-glutathionylation of cMyBPC (*S*-Glu-cMyBPC), protein lysates prepared from mouse ventricles were solubilized in a non-reducing 2× Laemmli buffer (Bio-Rad) as done previously.^13,14^ Without adding reducing agent and sample heating, proteins were separated on SDS-PAGE gels for standard Western blotting. For other protein targets, 2-mercaptoethanol (2.5% final) was applied to the protein lysates and samples were heated (95⁰C for 5 min) for denaturation. Proteins were separated on SDS-PAGE gels and transferred onto methanol-activated polyvinylidene difluoride membrane for 2 h at 65 mV and 4⁰C. After blocking (5% nonfat milk or 5% bovine serum albumin in Tris-buffered saline, 0.1% Tween 20) for 1 h, the membranes were incubated with the primary antibodies overnight at 4⁰C. Primary antibodies were purchased from Santa Cruz Biotechnology (Dallas, TX), Alomone Labs (Jerusalem, Israel), Cell Signaling Technology (Danvers, MA), or Virogen (Watertown, MA). Horseradish peroxidase-conjugated goat anti-rabbit and anti-mouse IgG secondary antibodies (Bio-Rad, Hercules, CA) were used with dilution of 1:4000 for 1 h at room temperature. Vinculin (Cell Signaling Technology), GAPDH or α-tubulin, or β-actin (Abcam) were used as loading controls. Optical density of the bands was measured with ChemiDoc MP System (Bio-Rad) and analyzed with Image Lab 6.0.0 software (Bio-Rad).

Isolation of ventricular cardiomyocytes. Mouse ventricular cardiomyocytes were isolated as described before.^11,15,16^ Briefly, hearts were excised under isoflurane (2%) anesthesia and perfused with buffer (mmol/L: 113 NaCl, 4.7 KCl, 0.6 Na_2_HPO_4_, 0.6 KH_2_PO_4_, 1.2 MgSO_4_, 0.032 Phenol Red, 12 NaHCO_3_, 10 KHCO_3_, 10 HEPES, 30 Taurine, 10 2-3-butanedione monoxime) for 7 min at 3 mL/min flow rate. Then, hearts were perfused with collagenase II (0.8 mg/mL, Worthington Biochemical Co. Lakewood, NJ) for 10 min at 37°C using a temperature controlled Langendorff perfusion system. The atria were removed. Following suspension with serially increasing Ca^2+^ concentrations (0.2, 0.5, and 1 mmol/L), cardiomyocytes were suspended immediately in standard Tyrode’s solution (containing in mmol/L: 140 NaCl, 5.4 KCl, 1 MgCl_2_, 10 HEPES, 1.8 CaCl_2_, and 5.5 glucose, pH 7.4).

### Mitochondrial ROS and intracellular pH measurement. mitoROS and intracellular pH were measured by confocal imaging as described before.^1,15,16^ Briefly, freshly isolated cardiomyocytes (recovered in an incubator for 1 h) or RL-14 cells were plated on glass-bottom MatTek 35-mm dishes. Cells were treated with MitoSOX Red (1 µmol/L, Thermo Fisher Scientific) in an incubator for 15 min or with pHrodo Green (5 µmol/L, Thermo Fisher Scientific) in an incubator for 30 min. Cells were washed twice with standard Tyrode’s solution to remove unbound fluorescent dyes. Confocal imaging was obtained with an Olympus FLUOVIEW FV3000 HSU confocal microscope (Evident Scientific, Waltham, MA) at excitation/emission of 488/560 nm or 518/540. Z-stack mean fluorescent intensity (MFI) was obtained with ImageJ-FIJI. The ΔMFI was obtained from the subtraction between the MFI of the cells and the background of the same images.

### Intracellular Mg^2+^ levels. Intracellular free Mg^2+^ was measured using a specific Mg^2+^ probe, Mag-fluo-4 AM (10 µmol/L, Thermo Fisher Scientific, Eugene, OR) by fluorescence confocal imaging by following the manufacturer’s guide. Briefly, RL-14 cardiomyocytes plated on glass-bottom MatTek 35-mm dishes (1.5 coverglass bottom, 0.16-0.19mm, Ashland, MA) were loaded with Mag-fluo-4 AM for 40 min followed by washing three times with Tyrode’s solution containing no Mg^2+^. This was followed by de-esterification of intracellular acetoxymethyl esters at room temperature for 30 min. Cell fluorescent intensity was recorded at excitation/emission of 488/520 nm at different extracellular Mg^2+^ concentrations (0, 0.04, 0.81, or 10 mmol/L) with Olympus FLUOVIEW FV3000 HSU Confocal (Evident Scientific, Waltham, MA). Intracellular Mg^2+^ level was evaluate with (F – F_min_)/(F_max_ – F): F=ΔMFI at 0.04 or 0.81 mmol/L extracellular Mg^2+^ for cell treated with low-Mg media or normal media, respectively, F_min_ = ΔMFI at 0 extracellular Mg^2+^, and F_max_ = ΔMFI at 10 mmol/L extracellular Mg^2+^; Z-stack ΔMFI was obtained with ImageJ-FIJI with subtraction of the background MFI of the same images. Data were then normalized to the control group.

SGLT2 protein detection with Mass spectrometry. Protein samples immunoprecipitated with SGLT2 antibody (Santa Cruz) were run on the SDS-PAGE gel, stained with EZBlue Gel Staining reagent, excised and sent to the Center for metabolomics and Proteomics, University of Minnesota for mass spectrometry. Briefly, excised gel regions were subjected to in-gel trypsin digestion as described previously (PubMed ID: 27134171) with the following change. During the alkylation step, 55 mmol/L iodoacetamide was used instead of 55 mmol/L methyl methanethiosulfonate. Post digestion, each sample was cleaned with a C18-like Stage tip (PMID: 12585499). Eluates were vacuum dried**.** The dried peptides were reconstituted in 97.9:2:0.1, H_2_O:acetonitrile (ACN):formic acid (FA) (load solvent) and analyzed approximately 100 ng of each sample by capillary LC-MS with a Thermo Fisher Scientific, Inc (Waltham, MA) Dionex UltiMate 3000 RSLCnano system on-line with an Orbitrap Eclipse mass spectrometer (Thermo Scientific, Waltham MA). The peptides were injected directly in load solvent and performed gradient separation on a self-packed C18 column (Dr. Maisch GmbH ReproSil-PUR 1.9 um 120 Å C18aq , 100 μm ID × 45 cm length) at 55°C with the following profile: 5% B solvent from 0 – 2 min, 8% B at 2.5 min, 21% B at 30 min, 35% B at 45 min and 90%B at 47 min with a flowrate of 350 nl/min from 0 – 2 min and 315 nl/minute from 2.5 – 60 min, where solvent A was 0.1% formic acid in water and solvent B was 0.1% formic acid in ACN. We employed a data independent acquisition (DIA) workflow on the Orbitrap with the following MS parameters: ESI voltage +2.1 kV, ion transfer tube 275 °C; no internal calibration; Orbitrap MS1 scan 60k resolution in profile mode from 390 – 990 *m/z* with 50 ms injection time; 100% (4 x 10E5) automatic gain control (AGC); we acquired tandem MS with HCD (high energy collision dissociation) activation at a fixed collision energy of 33% and orbitrap detection at 30k resolution; the precursor isolation window was set to 12 Da with a 2 Da overlap; the number of MS2 scan events was 75 per cycle and the MS2 loop count was set to 25; the MS2 injection time was 54 ms and the automatic gain control was set to 800%. For database searching, we processed peptide tandem MS using CHIMERYS 3.0.0 (https://www.msaid.de/) spectral prediction algorithm module in Proteome Discoverer 3.2 (Thermo Scientific). The *Mus musculus* Universal Proteome database (UP00000589) was downloaded from UniProt.org on August 20, 2024, merged with a common lab contaminant protein database (<https://github.com/HaoGroup-ProtContLib>) and used as the target database. The CHIMERYS search parameters were: enzyme trypsin full specificity, 1 missed cleave site, peptide length 7 – 30 amino acids, precursor charge 1 – 6, fragment ion mass tolerance 20 ppm, all feature traces were stored, and one job was processed for all files. We specified CAM cysteine (+57.021 Da) as a static modification and oxidation of methionine (+15.995 Da) as a dynamic modification (maximum 3 dynamic modifications per peptide). For the criteria for the protein identification, we applied 1% protein and peptide False Discovery Rate (FDR) and 2 peptide minimum as filters on the protein report using the Percolator algorithm^17^ in PD.

**Supplementary Figures**

**Supplementary Figure 1. HypoMg or Dapa treatment does not affect (A) systolic function (ejection fraction - EF%) or (B) the ratio of heart weight to body weight (HW/BW) in mice.** Control mice were fed with normal diet (2 g/kg Mg^2+^) and HypoMg mice with the low-Mg diet (15-30 mg/kg Mg^2+^ and ddH_2_O as drinking water) for 6 weeks starting at 10-week-old. Oral Dapa treatment (1.5 mg/kg/day) started after 3 weeks of HypoMg diet in half of the HypoMg group and lasted for 3 weeks. Seven to 25 mice were tested for each group. About half male and half female mice were tested in each group, except for the HypoMg mice that were mainly male mice. Red bars in figures are mean±SD. One-way ANOVA with Tukey’s multiple comparisons test was used for statistical analysis.

**Supplementary Figure 2. HypoMg decreased the mitochondrial antioxidant manganese superoxide dismutase (MnSOD) in mouse ventricular tissue, which was not recovered by Dapa treatment.** Control mice were fed with normal diet (2 g/kg Mg^2+^) and HypoMg mice with the low-Mg diet (15-30 mg/kg Mg^2+^ and ddH_2_O as drinking water) for 6 weeks starting at 10-week-old. Oral Dapa treatment (1.5 mg/kg/day) started after 3 weeks of HypoMg diet in half of the HypoMg group and lasted for 3 weeks. Four mice were tested for each group. About half male and half female mice were tested in each group, except for the HypoMg mice that were mainly male mice. Red bars in figures are mean±SD. One-way ANOVA with Tukey’s multiple comparisons test was used for statistical analysis. *P<0.05, **P<0.01.

**Supplementary Figure 3. NHE1 protein levels are increased by HypoMg in mouse ventricles and are unchanged by Dapa treatment.** Control mice were fed with normal diet (2 g/kg Mg^2+^) and HypoMg mice with the low-Mg diet (15-30 mg/kg Mg^2+^ and ddH_2_O as drinking water) for 6 weeks starting at 10-week-old. Oral Dapa treatment (1.5 mg/kg/day) started after 3 weeks of HypoMg diet in half of the HypoMg group and lasted for 3 weeks. Four mice were tested for each group. About half male and half female mice were tested in each group, except for the HypoMg mice that were mainly male mice. Protein molecular weight is labeled in kDa. Red bars in figures are mean±SD. One-way ANOVA with Tukey’s multiple comparisons test was used for statistical analysis, *P<0.05.

**Supplementary Figure 4. NHE1 activation with rosmarinic acid (RA) decreases intracellular free Mg^2+^ levels in RL-14 cardiomyocytes, and Dapa reverses the changes.** Human RL-14 cardiomyocytes were treated with rosmarinic acid (RA, 10 μg/mL) in the absence or presence of Dapa (5 µmol/L) in a normal medium (0.81 mmol/L Mg^2+^) for 48 h. Intracellular Mg^2+^ was measured with Mag-Fluo-4 AM fluorescence by confocal imaging; 35 to 42 cells from 3 dishes were tested for each group. Data were normalized to the control group. Red bars in figures are mean±SD. One-way ANOVA with Tukey’s multiple comparisons test was used for statistical analysis. *P<0.05, ***P<0.0001.

**References**

1	Liu, M. *et al.* Magnesium deficiency causes a reversible, metabolic, diastolic cardiomyopathy. *J Am Heart Assoc* **10**, e020205, doi:doi:10.1161/JAHA.120.020205 (2021).

2	Rude, R. K., Gruber, H. E., Wei, L. Y., Frausto, A. & Mills, B. G. Magnesium deficiency: effect on bone and mineral metabolism in the mouse. *Calcif. Tissue Int.* **72**, 32-41, doi:10.1007/s00223-001-1091-1 (2003).

3	Groenestege, W. M., Hoenderop, J. G., van den Heuvel, L., Knoers, N. & Bindels, R. J. The epithelial Mg^2+^ channel transient receptor potential melastatin 6 is regulated by dietary Mg^2+^ content and estrogens. *J. Am. Soc. Nephrol.* **17**, 1035-1043, doi:10.1681/asn.2005070700 (2006).

4	de Baaij, J. H. *et al.* Identification of SLC41A3 as a novel player in magnesium homeostasis. *Sci Rep* **6**, 28565, doi:10.1038/srep28565 (2016).

5	Leng, W. *et al.* The SGLT-2 Inhibitor Dapagliflozin Has a Therapeutic Effect on Atherosclerosis in Diabetic ApoE(-/-) Mice. *Mediators Inflamm* **2016**, 6305735, doi:10.1155/2016/6305735 (2016).

6	Wu, Q. *et al.* Dapagliflozin protects against chronic heart failure in mice by inhibiting macrophage-mediated inflammation, independent of SGLT2. *Cell Rep Med* **4**, 101334, doi:10.1016/j.xcrm.2023.101334 (2023).

7	Wei, R. *et al.* Dapagliflozin promotes beta cell regeneration by inducing pancreatic endocrine cell phenotype conversion in type 2 diabetic mice. *Metabolism* **111**, 154324, doi:10.1016/j.metabol.2020.154324 (2020).

8	Zaibi, N., Li, P. & Xu, S. Z. Protective effects of dapagliflozin against oxidative stress-induced cell injury in human proximal tubular cells. *PLoS One* **16**, e0247234, doi:10.1371/journal.pone.0247234 (2021).

9	Teshima, Y., Akao, M., Jones, S. P. & Marbán, E. Cariporide (HOE642), a selective Na^+^-H^+^ exchange inhibitor, inhibits the mitochondrial death pathway. *Circulation* **108**, 2275-2281, doi:10.1161/01.cir.0000093277.20968.c7 (2003).

10	Jung, S. W. *et al.* Rosmarinic Acid, as an NHE1 Activator, Decreases Skin Surface pH and Improves the Skin Barrier Function. *Int J Mol Sci* **23**, doi:10.3390/ijms23073910 (2022).

11	Liu, M. *et al.* Magnesium supplementation improves diabetic mitochondrial and cardiac diastolic function. *JCI Insight* **4**, e123182, doi:10.1172/jci.insight.123182 (2019).

12	Jeong, E. M. *et al.* Tetrahydrobiopterin improves diastolic dysfunction by reversing changes in myofilament properties. *J Mol Cell Cardiol* **56**, 44-54 (2013).

13	Lovelock, J. D. *et al.* Ranolazine improves cardiac diastolic dysfunction through modulation of myofilament calcium sensitivity. *Circ Res* **110**, 841-850, doi:CIRCRESAHA.111.258251 [pii];10.1161/CIRCRESAHA.111.258251 [doi] (2012).

14	Silberman, G. A. *et al.* Uncoupled cardiac nitric oxide synthase mediates diastolic dysfunction. *Circulation* **121**, 519-528, doi:CIRCULATIONAHA.109.883777 [pii];10.1161/CIRCULATIONAHA.109.883777 [doi] (2010).

15	Liu, M. *et al.* Mitochondrial dysfunction causing cardiac sodium channel downregulation in cardiomyopathy. *J Mol Cell Cardiol* **54**, 25-34, doi:10.1016/j.yjmcc.2012.10.011 (2013).

16	Liu, M. *et al.* Role of protein kinase C in metabolic regulation of the cardiac Na^+^ channel. *Heart Rhythm* **14**, 440-447 (2017).

17	Käll, L., Canterbury, J. D., Weston, J., Noble, W. S. & MacCoss, M. J. Semi-supervised learning for peptide identification from shotgun proteomics datasets. *Nat Methods* **4**, 923-925, doi:10.1038/nmeth1113 (2007).
